# Use of a Biostimulant to Mitigate the Effects of Excess Salinity in Soil and Irrigation Water in Tomato Plants

**DOI:** 10.3390/plants12051190

**Published:** 2023-03-06

**Authors:** Javier Zuzunaga-Rosas, Sara González-Orenga, Roberta Calone, Raúl Rodríguez-Heredia, Ali Asaff-Torres, Monica Boscaiu, Sara Ibáñez-Asensio, Héctor Moreno-Ramón, Oscar Vicente

**Affiliations:** 1 Department of Plant Production, Universitat Politècnica de València, Camino de Vera s/n, 46022 Valencia, Spainhecmora@prv.upv.es (H.M.-R.); 2 Innovak Global S. A. de C. V., Blvd. Lombardo Toledano 6615, La Concordia, Chihuahua 31375, Mexico; 3 Department of Plant Biology and Soil Science, Universidad de Vigo, Campus Lagoas-Marcosende, 36310 Vigo, Spain; 4 Institute for the Conservation and Improvement of Valencian Agrodiversity (COMAV), Universitat Politècnica de València, Camino de Vera s/n, 46022 Valencia, Spain; 5 Council for Agricultural Research and Economics (CREA), Research Centre for Agriculture and Environment, I-40128 Bologna, I-00184 Rome, Italy; 6 Centro de Investigación en Alimentación y Desarrollo (CIAD), A. C. Carretera Gustavo Enrique Astiazarán Rosas No. 46, La Victoria, Hermosillo 83304, Mexico; 7 Mediterranean Agroforestry Institute (IAM), Universitat Politècnica de València, Camino de Vera s/n, 46022 Valencia, Spain

**Keywords:** salt stress, oxidative stress, oxidative stress markers, ion transport, salinity tolerance, antioxidant systems, osmolytes, glycine betaine, polyphenols

## Abstract

Global warming is linked to progressive soil salinisation, which reduces crop yields, especially in irrigated farmland on arid and semiarid regions. Therefore, it is necessary to apply sustainable and effective solutions that contribute to enhanced crop salt tolerance. In the present study, we tested the effects of a commercial biostimulant (BALOX^®^) containing glycine betaine (GB) and polyphenols on the activation of salinity defense mechanisms in tomato. The evaluation of different biometric parameters and the quantification of biochemical markers related to particular stress responses (osmolytes, cations, anions, oxidative stress indicators, and antioxidant enzymes and compounds) was carried out at two phenological stages (vegetative growth and the beginning of reproductive development) and under different salinity conditions (saline and non-saline soil, and irrigation water), using two formulations (different GB concentrations) and two doses of the biostimulant. Once the experiments were completed, the statistical analysis revealed that both formulations and doses of the biostimulant produced very similar effects. The application of BALOX^®^ improved plant growth and photosynthesis and assisted osmotic adjustment in root and leaf cells. The biostimulant effects are mediated by the control of ion transport, reducing the uptake of toxic Na^+^ and Cl^−^ ions and favoring the accumulation of beneficial K^+^ and Ca^2+^ cations, and a significant increase in leaf sugar and GB contents. BALOX^®^ significantly reduced salt-induced oxidative stress and its harmful effects, as evidenced by a decrease in the concentration of oxidative stress biomarkers, such as malondialdehyde and oxygen peroxide, which was accompanied by the reduction of proline and antioxidant compound contents and the specific activity of antioxidant enzymes with respect to the non-treated plants.

## 1. Introduction

Currently, there is compelling scientific evidence that climatic changes have the potential to dramatically alter biodiversity in nature [1] and decrease crop yields [2]. The population of the globe is predicted to keep growing over the next three decades, reaching roughly 10 × 10^9^ people by 2050 [3]. Future food security is undoubtedly at risk from extreme environmental stresses brought on by climate change.

The most problematic environmental factors driving agricultural losses are drought and soil salinisation [4]. Water shortages are viewed as a significant worldwide issue that directly affects agricultural systems, and climate projections indicate that they will worsen in the coming decades [5]. Due to its antagonistic effects on nutrient uptake and transport within plants, salinity can impair their nutritional balance [6], significantly reducing crop yields [7]. Along with other natural soil salinity factors, over-irrigation with poor-quality, salinised water intensifies this problem [8]. Nowadays, salt has some impact on more than 10^9^ hectares of the global land area, and salinised soils are typically expanding at a rate of 80.000 to 100.000 km^2^ each year [9]. Via pleiotropic processes including osmotic stress, ionic toxicity, nutritional imbalance, and oxidative stress, salinity negatively affects plant growth, development, and productivity [10].

Most horticultural crops are sensitive to salinity and grow poorly in salinised soils. In this situation, an accumulation of ions occurs due to continued irrigation [11]. The tomato (*Solanum lycopersicum* L.) is one of the most important vegetable crops. It is relatively tolerant to mild salt stress but sensitive to high salinity; it has a wide range of cultivars adapted to different growing circumstances in different climatic zones, both in the open field and in greenhouses. Its cultivated area, according to FAOSTAT, exceeds 4.7 million ha globally, producing more than 182 million tonnes of fruit [12]. Due to its exceptional nutritional value, which includes being a great source of antioxidants, dietary fibre, minerals, and vitamins, as well as providing lycopene, phytoalexins, phenolic compounds, protease inhibitors, glycoalkaloids, and carotenoids, amongst other metabolites [13,14], its fruit is regarded as a safe and protective food. These antioxidants are highly effective at scavenging dangerous oxygen free radicals, enhancing night vision, guarding against tumours, ageing, and other health problems [15]. Consuming tomatoes has been linked to lowering blood pressure, preventing cancer, and controlling weight [16]. It has only 18 kilocalories per 100 g and contains very low fat and cholesterol levels [15].

The tomato physiology is negatively impacted by salt stress, which has an adverse effect on growth and production [17]. In addition, during their early development phases, tomato plants are particularly vulnerable to saline stress [18].

During the early phases of vegetative growth, salt stress is extremely problematic for many crops of global importance. Yields can be significantly reduced by electrical conductivity in irrigation water (ECw) higher than 1.5 dS/m [19]. The tomato plant is moderately susceptible to salt stress, as it may withstand ECw of 1.7 dS/m or ECe (saturated paste EC) in the soil of 2.5 dS/m without any significant yield reduction [11]. However, above this threshold value, tomato yield can decrease between 9% and 11% for each ECw unit, causing yield losses of 10% to 80% [7,9]. Salinity stress causes a reduction in a plant’s ability to absorb water from its roots, which lowers the plant’s water content (WC) and its fresh weight (FW). Additionally, changes in osmotic pressure, functional membrane integrity, nutrient balance, and redox homeostasis impact plants, resulting in a decrease in plant height, leaf number, or chlorophyll content [20,21,22,23].

Developing new crop varieties with enhanced resistance to abiotic stress through conventional breeding and genetic engineering or genome editing techniques would be the most sensible approach to mitigate the detrimental impacts of climate change on crop yields. We should be confident that this objective will be met for key crops fairly soon, thanks to several successful examples of both strategies [24]. However, there are currently several complementary approaches, such as the use of biostimulants, that can aid in enhancing the resilience of crops and reducing the effects of climate change [25]. The rise in scientific publications and the consistent growth of their market are indications that biostimulants are regarded as novel agricultural tools providing sustainable solutions to improve agriculture production. Regulations set forth by the European Commission define biostimulants as substances that promote a crop’s natural nutritional processes, such as improving nutrient availability in the rhizosphere, nutrient usage efficiency, abiotic stress tolerance, or crop quality traits [26]. Biostimulants are substances with a high concentration of bioactive components that are generally produced from natural sources. Even under adverse circumstances, they have been found to boost plant growth and productivity by stimulating physiological and metabolic processes [27]. Biostimulants, unlike fertilisers, do not directly provide nutrients to plants. Instead, they improve absorption and boost the effectiveness of nutrient usage, which minimises the need for fertiliser application [28]. Therefore, biostimulants can contribute to lowering the current large-scale application of mineral fertilisers. Until now, efforts to increase food production have resulted in a significant reliance on synthetic agrochemicals and non-renewable mineral fertilisers [29], a practice that is unsustainable in the long term.

Biostimulants are primarily derived from a variety of organic materials, including humic, fulvic, and carboxylic acids, protein hydrolysates, nitrogen-containing compounds, seaweed and microalgae extracts, vitamins, amino acids, ascorbic acid, phenolic compounds, and other substances. Their formulations, which can be monocomponent or multicomponent, can be successfully applied to horticultural and agricultural crops [30].

Understanding the precise mechanisms triggered by biostimulants in plants can be challenging due to their complexity. When applied to various crops, most commercially available biostimulants result in a wide variety of plant responses [31]. To prevent product waste, excessive production costs and unexpected outcomes, finding the proper biostimulant dosage is just as crucial as choosing the right application method, either foliar or in the soil. Studying the fundamental and cutting-edge concepts behind the effects and application of biostimulants in agriculture is a challenge and an excellent opportunity for researchers in this sector [32].

Plants, especially in abiotic stress conditions such as salinity and drought, produce a quaternary ammonium chemical substance named glycine betaine (GB) [33]. Its exogenous application has been successfully used for improving crop resistance to different environmental stresses [33,34,35,36], at least partly due to enhanced photosynthesis efficiency [37]. Polyphenols, on the other hand, are constituents of different biostimulants, which are reported to stimulate growth or stress tolerance in several plant species [38,39,40]. However, there are only a few reports on the possible synergetic effects of GB and polyphenols, and they are limited to post-harvest treatments [41].

In a previous study, BALOX^®^, a biostimulant based on GB and polyphenols, was tested on tomato plants and had a positive effect on plant growth, especially on the root development in saline soils [42]. It also partially inhibited the root uptake of Na^+^ and Cl^−^ ions and improved the accumulation of K^+^ and Ca^2+^ to some extent. In addition, it reduced oxidative stress, leaf MDA, H_2_O_2_ concentrations, and the specific activities of antioxidant enzymes. However, the mode of action of the biostimulant and its optimal application conditions have not yet been completely clarified.

The primary aim of the current research was to confirm and extend the already available data, including a complex set of additional variables of agronomic and practical relevance. The impact of different dosages of the biostimulant and a higher glycine betaine content in its composition was tested to check if this would result in enhanced salt tolerance in tomatoes. Furthermore, in contrast to the previous work, we used a loamy-textured agricultural soil with two levels of salinity as the substrate for plant growth and performing plant irrigation with saline and non-saline water.

The working hypothesis was that the biostimulant would enhance plant tolerance to increasing salt concentrations, improving the responses to osmotic, ionic, and oxidative stress, the three main components of salt stress. In addition, it should be possible to select the biostimulant’s most convenient dose and composition in terms of efficiency and economics. To reach this objective, we have analysed several plant biometric parameters and multiple biochemical stress markers, such as photosynthetic pigments, ion concentrations in roots and leaves, foliar contents of different osmolytes, oxidative stress markers, antioxidant compounds, and the specific activity of various antioxidant enzymes, in tomato plants subjected to the combination of treatments mentioned above during two growth periods: 30 days (plants in the vegetative development phase) and 60 days (plants at the onset of reproductive development).

## 2. Results

### 2.1. Exploratory Analysis of Experimental Factors

The experiments were based on a multivariate design that consisted of the use of a biostimulant (BALOX^®^) with a high content of polyphenols and glycine betaine, applied under two formulations: B1: concentrations of 1.4% and 3.0% (*w*/*w*) of polyphenols and glycine betaine, respectively, and B2: with a variation in the concentration of glycine betaine at 5.0% (*w*/*w*) and the same polyphenol content (1.4%). Two doses of the biostimulant were used (D1: 0.4 mL L^−1^ irrigation water and D2: 0.8 mL L^−1^ irrigation water). In addition, two soil conditions, non-saline (EC = 2.28 dS/m) and saline soil (EC = 8.55 dS/m), and two types of irrigation, with non-saline (EC = 0.91 dS/m) and with saline water (EC = 10.50 dS/m) were included in the experiments. The biostimulant was applied in Hoagland solution to each pot at the basal part of the plant. The treatments were extended for 30 days for plants in the vegetative phase of development and for 60 days for plants at the beginning of reproductive development. Control plants (Ctrl) were cultivated without biostimulants (D0: 0 mL L^−1^ irrigation water) while maintaining the same experimental design.

**Figure 1:**
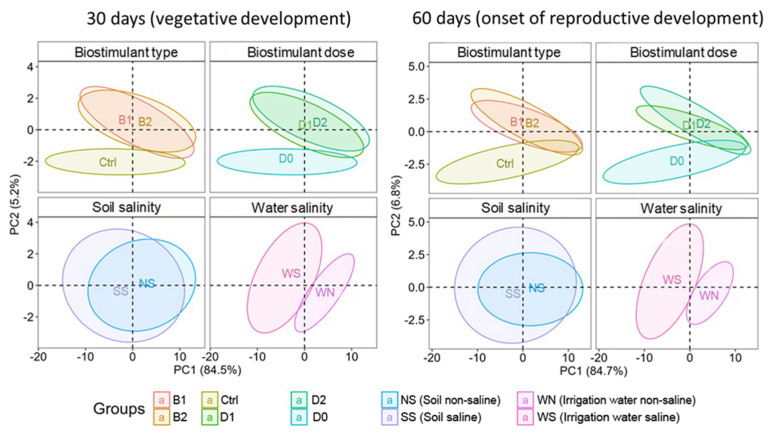
Principal component analysis (PCA). An interaction graph of the four experimental factors studied at 30 days in the vegetative developmental stage (left) and at 60 days, at the beginning of reproductive development (right), in *Solanum lycopersicum* plants. The factors studied were: Biostimulant type [Ctrl: control; B1: biostimulant with polyphenols at 1.4% (*w*/*w*) and glycine betaine at 3.0% (*w*/*w*); B2: biostimulant with polyphenols at 1.4% (*w*/*w*) and glycine betaine at 5.0% (*w*/*w*)]; Biostimulant dose (D0: 0 mL L^−1^ irrigation water; D1: 0.4 mL L^−1^ irrigation water; D2: 0.8 mL L^−1^ irrigation water); Soil salinity (Soil non-saline (NS): EC = 2.28 dS/m; Soil saline (SS): EC = 8.55 dS/m); Water salinity (Irrigation water non-saline (WN): EC = 0.91 dS/m; and Irrigation water saline (WS): EC = 10.50 dS/m).

Once all the treatments and analyses were concluded and all the data collected, an exploratory principal component analysis (PCA) (Figure 1) was performed to examine the four experimental factors (type of biostimulant, biostimulant dose, soil salinity, and water salinity) and their interaction with all the morphological and biochemical parameters determined in this study.

As seen in Figure 1, the overlap between the ellipses indicates the degree of similarity between the groups. The ellipses corresponding to levels B1 and B2 of the factor “Type of biostimulant” and levels D1 and D2 of the factor “Biostimulant dose” are largely overlapping, since they are very similar for each treatment period. On the contrary, there is minimal overlapping with the corresponding controls at 60 days and almost none at 30 days. Therefore, it could be assumed that the biostimulant effects are practically the same regardless of its glycine betaine content, 3% (B1) or 5% (B2). Likewise, the lower BALOX^®^ dose (D1, 0.4 mL L^−1^ irrigation water) was as effective as the double dose (D2).

Regarding the factor “Soil salinity”, there is also extensive overlapping between the ellipses corresponding to the two types of soil, although that of the saline soil is somewhat larger than that of the non-saline soil, especially after 60 days of treatment. This pattern is probably due to the accumulation of salts in the saline soil because of irrigation with saline water during the experiment. Considering the two levels of the “Water salinity” factor, there is a marked difference between the two ellipses, which do not overlap, with that of saline soil being much larger than the other, both after 30 and 60 days of treatment. This pattern supports the significantly different effects of irrigation with saline or non-saline water.

According to the PCA outcomes, both biostimulant compositions and the two doses have very similar effects. Therefore, it is unnecessary to show the results of all combined treatments. In the following sections, data will be presented for the control (Ctrl) and the biostimulant (B1) containing polyphenols at 1.4% (*w*/*w*) with the lowest concentration of glycine betaine (3%) at the lowest dose (D1, 0. 4 mL L^−1^ irrigation water), the two types of soil (i.e., non-saline and saline soil), and two irrigation conditions (i.e., with non-saline and saline water). The combination of these factors resulted in four levels of salinity, from non-saline to high salinity conditions: (i) NSWN: Non-saline soil (EC = 2.28 dS/m) and non-saline water (EC = 0.91 dS/m); (ii) SSWN: Saline soil (EC = 8.55 dS/m) and non-saline water (EC = 0.91 dS/m); (iii) NSWS: Non-saline soil (EC = 2.28 dS/m) and saline water (EC = 10.50 dS/m); and (iv) SSWS: saline soil (EC = 8.55 dS/m) and saline water (EC = 10.50 dS/m). Plants were evaluated in the vegetative phase of development after 30 days of treatment and at the beginning of the reproductive phase after 60 days of treatment.

### 2.2. Effect of the Treatments on Plant Growth

**Figure 2:**
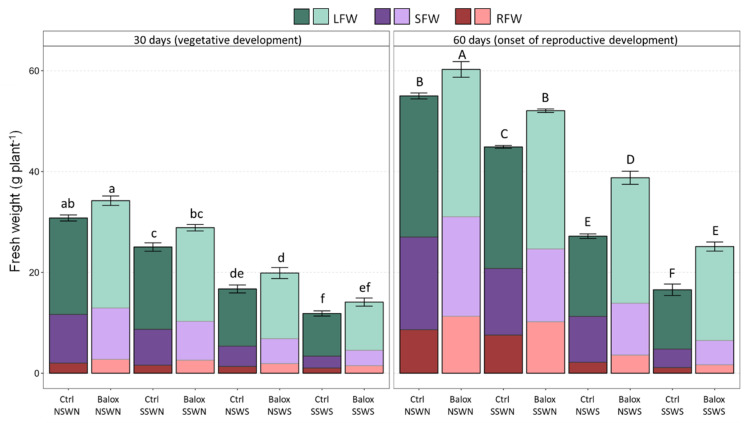
Total fresh weight, shown as the sum of root, stem, and leaf FW, of tomato plants subjected to increasing salinity by different combinations of saline or non-saline soil and irrigation water; the plants were treated, or not, with the biostimulant containing polyphenols at 1.4% (*w*/*w*) and glycine betaine at 3.0% (*w*/*w*) at a dose of 0.4 mL L^−1^ of irrigation water. Symbols: LFW, leaf fresh weight; SFW, stem fresh weight; RFW, root fresh weight. The control treatments without biostimulant (Ctrl) and with biostimulant (BALOX^®^) are defined as follows: (i) NSWN: Non-saline soil (EC = 2.28 dS/m) and non-saline water (EC = 0.91 dS/m); (ii) SSWN: Saline soil (EC = 8.55 dS/m) and non-saline water (EC = 0.91 dS/m); (iii) NSWS: Non-saline soil (EC = 2.28 dS/m) and saline water (EC = 10.50 dS/m); (iv) SSWS: Saline soil (EC = 8.55 dS/m) and saline water (EC = 10.50 dS/m). The evaluation periods: 30 days and 60 days of treatments. The values are the means ± SE (*n* = 3). Different letters above the bars (lowercase for 30 days and capital for 60 days treatments) indicate significant differences between treatments, according to the ANOVA test (*p* < 0.05). Statistical analyses were performed independently for the two treatment periods.

Salt stress significantly inhibited the growth of control (no biostimulant added) tomato plants in parallel with increasing salinity, as shown by the determination of several morphological parameters (Figure 2 and Supplementary Materials, Tables S1 and S2). Compared to the non-saline control (CtrlNSWN), a progressive decrease of fresh weight (FW) was observed for roots, stems, and leaves of plants grown under SSWN, NSWS, and SSWS conditions for 30 or 60 days, resulting in a statistically significant reduction of the total fresh plant weight. For example, at the onset of reproductive development after 60 days of growth, plant FW decreased by 18% upon irrigation with non-saline water in saline soil (SS), by 50% in non-saline soil with saline water (WS), and by 70% under the highest salinity conditions tested, saline soil and saline irrigation water (SSWS) (Figure 2). The different individual values for FW of leaves, stems, and roots are shown in Table S1 of the Supplementary Materials. Salt-induced growth inhibition was also reflected in the relative reduction with respect to the control of other growth parameters, such as plant height, stem diameter, and leaf number (Supplementary Materials, Table S2).

The application of the biostimulant enhanced plant growth under all experimental conditions tested. The mean values of all growth parameters (root, stem, and leaf FW, plant height, stem diameter, and leaf number) increased with respect to the corresponding controls without biostimulant, although these increments were statistically significant only in some circumstances (Supplementary Materials, Tables S1 and S2). Regarding whole plant FW, the increases detected in the presence of BALOX^®^ were significant for all treatments after the 60-day period but not at 30-day period (Figure 2). The relative increase in FW was more pronounced as salinity increased, ranging between 1.1-fold in the non-saline control (CtrlNSWN) and 1.5-fold for plants grown in the combination of saline soil and irrigated with saline water (SSWS).

**Figure 3:**
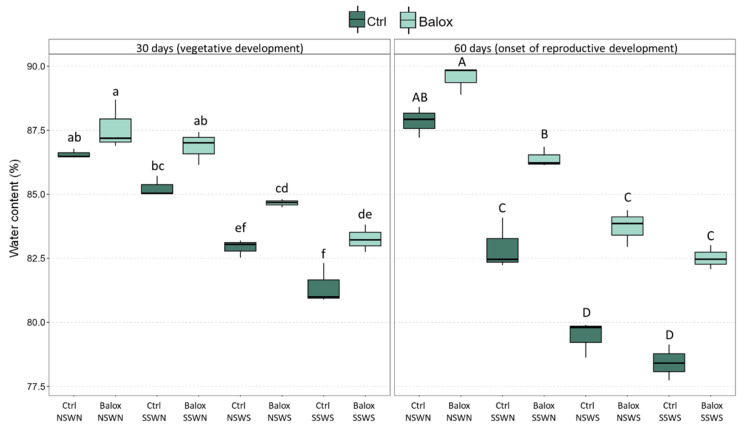
Total water content in tomato plants subjected to increasing salinity by different combinations of saline or non-saline soil and irrigation water; the plants were treated, or not, with the biostimulant containing polyphenols at 1.4% (*w*/*w*) and glycine betaine at 3.0% (*w*/*w*) at a dose of 0.4 mL L^−1^ of irrigation water. The control treatments without biostimulant (Ctrl) and with biostimulant (BALOX^®^) are defined as follows: (i) NSWN: Non-saline soil (EC = 2.28 dS/m) and non-saline water (EC = 0.91 dS/m); (ii) SSWN: Saline soil (EC = 8.55 dS/m) and non-saline water (EC = 0.91 dS/m); (iii) NSWS: Non-saline soil (EC = 2.28 dS/m) and saline water (EC = 10.50 dS/m); (iv) SSWS: Saline soil (EC = 8.55 dS/m) and saline water (EC = 10.50 dS/m). The evaluation periods: 30 days and 60 days of treatments. The values are the means ± SE (*n* = 3). Different letters above the bars (lowercase for 30 days and capital for 60 days treatments) indicate significant differences between treatments, according to the ANOVA test (*p* < 0.05). Statistical analyses were performed independently for the two treatment periods.

The salt-induced reduction of plant FW was, to a large extent, due to plant dehydration. The H_2_O content of leaves, stems, and roots decreased significantly in response to increasing salinity (Supplementary Materials, Table S3). Considering the whole plant, substantial drops in plant water content (WC) were observed, more pronounced at higher salinities and after the 60-day treatments (Figure 3). BALOX^®^ application resulted in all cases in a relative increase of mean WC, which was statistically significant under high salinity conditions (NSWS and SSWS) at 30 days and significant and more pronounced in all treatments after the 60-day period. Therefore, it appears that the biostimulant protects tomato plants against salt-induced dehydration.

### 2.3. Photosynthetic Pigment Contents

**Figure 4:**
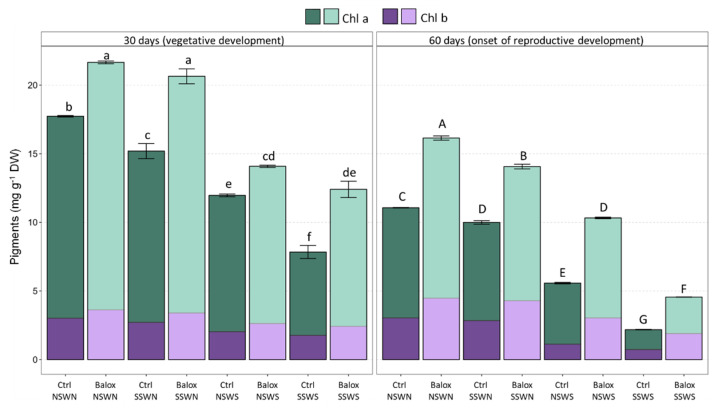
Total chlorophyll content in tomato plants subjected to increasing salinity by different combinations of saline or non-saline soil and irrigation water; the plants were treated, or not, with the biostimulant containing polyphenols at 1.4% (*w*/*w*) and glycine betaine at 3.0% (*w*/*w*) at a dose of 0.4 mL L^−1^ of irrigation water. The control treatments without biostimulant (Ctrl) and with biostimulant (BALOX^®^) are defined as follows: (i) NSWN: Non-saline soil (EC = 2.28 dS/m) and non-saline water (EC = 0.91 dS/m); (ii) SSWN: Saline soil (EC = 8.55 dS/m) and non-saline water (EC = 0.91 dS/m); (iii) NSWS: Non-saline soil (EC = 2.28 dS/m) and saline water (EC = 10.50 dS/m); (iv) SSWS: Saline soil (EC = 8.55 dS/m) and saline water (EC = 10.50 dS/m). The evaluation periods: 30 days and 60 days of treatments. The values are the means ± SE (*n* = 3). Different letters above the bars (lowercase for 30 days and capital for 60 days treatments) indicate significant differences between treatments, according to the ANOVA test (*p* < 0.05). Statistical analyses were performed independently for the two treatment periods.

**Figure 5:**
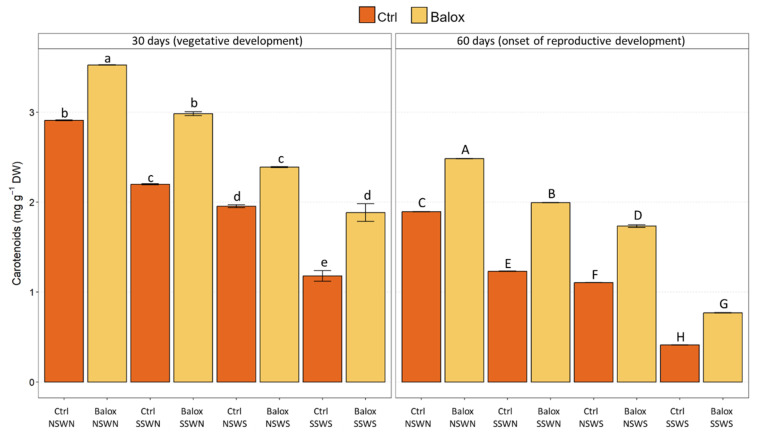
Carotenoids contents in tomato plants subjected to increasing salinity by different combinations of saline or non-saline soil and irrigation water; the plants were treated, or not, with the biostimulant containing polyphenols at 1.4% (*w*/*w*) and glycine betaine at 3.0% (*w*/*w*) at a dose of 0.4 mL L^−1^ of irrigation water. The control treatments without biostimulant (Ctrl) and with biostimulant (BALOX^®^) are defined as follows: (i) NSWN: Non-saline soil (EC = 2.28 dS/m) and non-saline water (EC = 0.91 dS/m); (ii) SSWN: Saline soil (EC = 8.55 dS/m) and non-saline water (EC = 0.91 dS/m); (iii) NSWS: Non-saline soil (EC = 2.28 dS/m) and saline water (EC = 10.50 dS/m); (iv) SSWS: Saline soil (EC = 8.55 dS/m) and saline water (EC = 10.50 dS/m). The evaluation periods: 30 days and 60 days of treatments. The values are the means ± SE (*n* = 3). Different letters above the bars (lowercase for 30 days and capital for 60 days treatments) indicate significant differences between treatments, according to the ANOVA test (*p* < 0.05). Statistical analyses were performed independently for the two treatment periods.

The mean values of total chlorophylls (Figure 4) and total carotenoids (Figure 5), showed a significant decreasing trend that can be related to the increasing salinity, being more pronounced at the end of the experiment (60 days). For example, total chlorophyll levels of control plants in non-saline conditions (CtrlNSWN) decreased by ca. 50% in plants grown for 60 d in non-saline soil and irrigated with saline water (CtrlNSWS), and by 80% under the highest salinity conditions tested, saline soil and saline irrigation (CtrlSSWS) (Figure 4). Separate data for chlorophylls *a* and *b* are shown in Table S4 of the Supplementary Materials. The relative reduction in total carotenoid contents with increasing salinity was similar to that determined for chlorophylls (Figure 5). Application of the biostimulant BALOX^®^ resulted in a statistically significant increase in chlorophylls *a* and *b* (Supplementary Materials, Table S4), total chlorophyll (Figure 4), and total carotenoid (Figure 5) contents. Here again, the relative effect of the biostimulant was more pronounced after 60 d of treatments and at higher salinities. For example, total chlorophyll contents increased about 1.5-fold in tomato plants grown for 60 d under non-saline conditions and 2.1-fold in saline soil with saline irrigation (Figure 4).

### 2.4. Ion Accumulation

**Figure 6:**
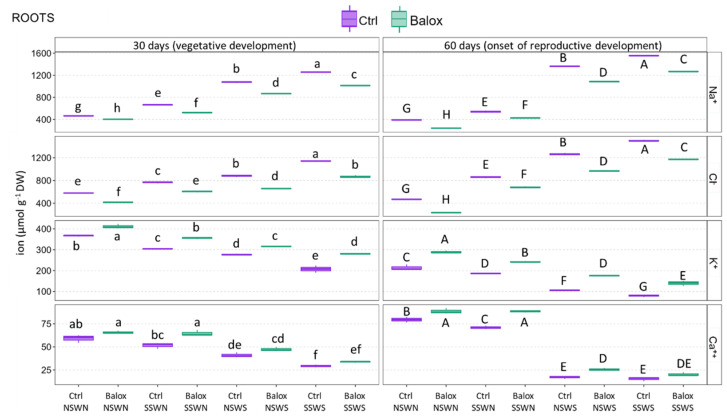
Ion contents in roots: sodium (Na^+^), chloride (Cl^−^), potassium (K^+^), and calcium (Ca^2+^) in tomato plants subjected to increasing salinity by different combinations of saline or non-saline soil and irrigation water; the plants were treated, or not, with the biostimulant containing polyphenols at 1.4% (*w*/*w*) and glycine betaine at 3.0% (*w*/*w*) at a dose of 0.4 mL L^−1^ of irrigation water. The control treatments without biostimulant (Ctrl) and with biostimulant (BALOX^®^) are defined as follows: (i) NSWN: Non-saline soil (EC = 2.28 dS/m) and non-saline water (EC = 0.91 dS/m); (ii) SSWN: Saline soil (EC = 8.55 dS/m) and non-saline water (EC = 0.91 dS/m); (iii) WNWS: Non-saline soil (EC = 2.28 dS/m) and saline water (EC = 10.50 dS/m); (iv) SSWS: Saline soil (EC = 8.55 dS/m) and saline water (EC = 10.50 dS/m). The evaluation periods: 30 days and 60 days of treatments. The values are the means ± SE (*n* = 3). Different letters above the bars (lowercase for 30 days and capital for 60 days of treatments) indicate significant differences between treatments, according to the ANOVA test (*p* < 0.05). Statistical analyses were performed independently for the two treatment periods.

Sodium (Na^+^) and chloride (Cl^−^) ion content increased significantly in the roots (Figure 6) of tomato plants in response to higher salinity. A maximum concentration was reached in plants that were irrigated with salted water and grown on salty soils (Ctrl SSWS): about 1200 µmol g^−1^ DW after 30 d of treatment, and 1500 µmol g^−1^ DW at the 60-day harvest, for both ions. In the 30-day treatment, these changes represented an increase of 2.7-fold for Na^+^ and 2.0-fold for Cl^−^ with respect to the non-saline conditions; the relative increment of Na^+^ and Cl^−^ root concentrations at 60 days was higher, about 3.9- and 3.2-fold, respectively (Figure 6). The consequence of BALOX^®^ use was clear: a significant reduction of the concentrations of both ions under all experimental conditions tested, in the range of 15–25% at 30 days and somewhat higher after 60 days of treatment (Figure 6).

Contrary to Na^+^ and Cl^−^, root K^+^ contents progressively decreased with increasing salinity, down to ca. 200 µmol g^−1^ DW, 56% of the non-saline control (CtrlNSWN), after 30 days of growth in saline soil with saline irrigation (SSWS). The absolute K^+^ concentrations were lower after 60 days of treatment under all experimental conditions, but the relative salinity-dependent reduction of its contents was more pronounced (down to 37% of the control). The addition of the biostimulant resulted in a statistically significant increase of K^+^ contents in roots under all tested conditions, which was also more pronounced at 60 days and up to 1.7-fold higher than in the absence of BALOX^®^ (Figure 6).

Finally, root Ca^2+^ contents also decreased progressively in parallel to increasing salinity, from ca. 60 to 34 µmol g^−1^ DW in the 30-day harvest and from 80 to 20 µmol g^−1^ DW after 60 days. In biostimulant-treated plants, the mean Ca^2+^ concentrations increased in all treatments, between 1.1- and 1.5-fold, although the differences with the non-treated plants were not significant at 30 days (Figure 6).

**Figure 7:**
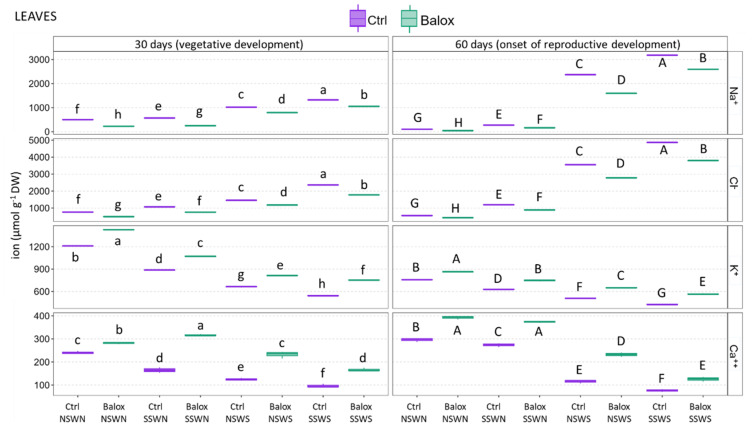
Ion contents in leaves: sodium (Na^+^), chloride (Cl^−^), potassium (K^+^), and calcium (Ca^2+^) in tomato plants subjected to increasing salinity by different combinations of saline or non-saline soil and irrigation water; the plants were treated, or not, with the biostimulant containing polyphenols at 1.4% (*w*/*w*) and glycine betaine at 3.0% (*w*/*w*) at a dose of 0.4 mL L^−1^ of irrigation water. The control treatments without biostimulant (Ctrl) and with biostimulant (BALOX^®^) are defined as follows: (i) NSWN: Non-saline soil (EC = 2.28 dS/m) and non-saline water (EC = 0.91 dS/m); (ii) SSWN: Saline soil (EC = 8.55 dS/m) and non-saline water (EC = 0.91 dS/m); (iii) NSWS: Non-saline soil (EC = 2.28 dS/m) and saline water (EC = 10.50 dS/m); (iv) SSWS: Saline soil (EC = 8.55 dS/m) and saline water (EC = 10.50 dS/m). The evaluation periods: 30 days and 60 days of treatments. The values are the means ± SE (*n* = 3). Different letters above the bars (lowercase for 30 days and capital for 60 days of treatments) indicate significant differences between treatments, according to the ANOVA test (*p* < 0.05). Statistical analyses were performed independently for the two treatment periods.

The patterns of variation of ion concentrations were qualitatively identical in leaves (Figure 7) and roots (Figure 6). That is, significant and progressive increases in Na^+^ and Cl^−^ leaf contents were observed with increasing salinity, both in the 30- and 60-day treatments, and, under all experimental conditions, BALOX^®^ application caused a significant reduction of the two toxic ions. Conversely, K^+^ and Ca^2+^ leaf contents decreased significantly with rising salinity and increased in all treatments in the presence of the biostimulant. The absolute concentrations of all ions were generally higher in leaves than in roots. For example, Na^+^ and Cl^−^ reached maximum concentrations in leaves of 3180 and 4870 µmol g^−1^ DW, respectively, compared to ca. 1500 µmol g^−1^ DW in roots for both ions (Ctrl SSWS, 60 d). Maximum K^+^ concentrations without biostimulant were 1200 µmol g^−1^ DW in leaves and ca. 370 µmol g^−1^ DW in roots (CtrlNSWN, 30 d), whereas the corresponding values for Ca^2+^ were ca. 300 and 80 µmol g^−1^ DW, respectively (CtrlNSWN, 60 d) (Figure 7).

### 2.5. Osmolytes Contents

**Figure 8:**
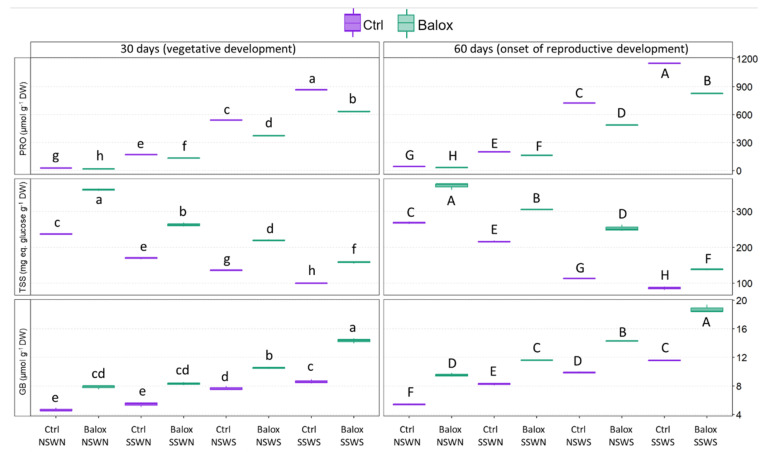
Proline (PRO), glycine betaine (GB), and total soluble sugars (TSS) content in leaves of tomato plants subjected to increasing salinity by different combinations of saline or non-saline soil and irrigation water; the plants were treated, or not, with the biostimulant containing polyphenols at 1.4% (*w*/*w*) and glycine betaine at 3.0% (*w*/*w*) at a dose of 0.4 mL L^−1^ of irrigation water. The control treatments without biostimulant (Ctrl) and with biostimulant (BALOX^®^) are defined as follows: (i) NSWN: Non-saline soil (EC = 2.28 dS/m) and non-saline water (EC = 0.91 dS/m); (ii) SSWN: Saline soil (EC = 8.55 dS/m) and non-saline water (EC = 0.91 dS/m); (iii) NSWS: Non-saline soil (EC = 2.28 dS/m) and saline water (EC = 10.50 dS/m); (iv) SSWS: Saline soil (EC = 8.55 dS/m) and saline water (EC = 10.50 dS/m). The evaluation periods: 30 days and 60 days of treatments. The values are the means ± SE (*n* = 3). Different letters above the bars (lowercase for 30 days and capital for 60 days of treatments) indicate significant differences between treatments, according to the ANOVA test (*p* < 0.05). Statistical analyses were performed independently for the two treatment periods.

Figure 8 shows the effects of the different treatments on the leaf levels of the three most common plant osmolytes: proline (Pro), total soluble sugars (TSS), and glycine betaine (GB). In the absence of the product, Pro levels increased progressively in response to increasing salinity, up to ca. 30-fold over the concentrations measured under non-saline conditions, reaching a maximum value of 1150 µmol g^−1^ DW (CtrlSSWS, 60 days). Under all experimental conditions, adding the biostimulant significantly reduced Pro levels (Figure 8).

The pattern of variation in TSS contents was just the opposite of that of Pro. A significant decrease was observed with increasing salinity, down to ca. 70% of the “no salinity” control (CtrlNSWN) after 30 days and 50% at 60 days of treatment. BALOX^®^ application resulted, in all cases, in a significant increase in TSS levels, from 1.5- to 2-fold with respect to the plants not treated with the biostimulant (Figure 8).

Leaf GB concentrations also increased significantly with salinity, even more so in the presence of BALOX^®^, although this was to be expected as it contains GB. Nevertheless, the absolute concentrations of this osmolyte, reaching maximum values below 20 µmol g^−1^ DW, are too low to have any relevant osmotic effect in stressed plants (Figure 8).

### 2.6. Oxidative Stress Markers

**Figure 9:**
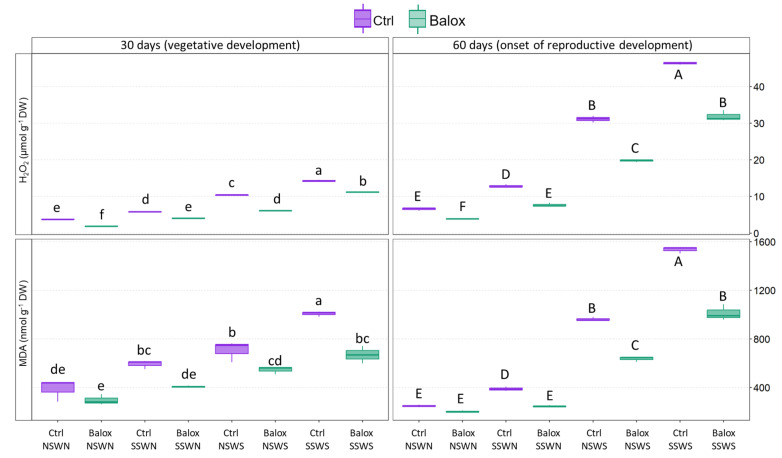
Hydrogen peroxide (H_2_O_2_) and malondialdehyde (MDA) contents in leaves of tomato plants subjected to increasing salinity by different combinations of saline or non-saline soil and irrigation water; the plants were treated, or not, with the biostimulant containing polyphenols at 1.4% (*w*/*w*) and glycine betaine at 3.0% (*w*/*w*) at a dose of 0.4 mL L^−1^ of irrigation water. The control treatments without biostimulant (Ctrl) and the treatments with biostimulant (BALOX^®^) are defined as follows: (i) NSWN: Non-saline soil (EC = 2.28 dS/m) and non-saline water (EC = 0.91 dS/m); (ii) SSWN: Saline soil (EC = 8.55 dS/m) and non-saline water (EC = 0.91 dS/m); (iii) NSWS: Non-saline soil (EC = 2.28 dS/m) and saline water (EC = 10.50 dS/m); (iv) SSWS: Saline soil (EC = 8.55 dS/m) and saline water (EC = 10.50 dS/m). The evaluation periods: 30 days and 60 days of treatments. The values are the means ± SE (*n* = 3). Different letters above the bars (lowercase for 30 days and capital for 60 days of treatments) indicate significant differences between treatments, according to the ANOVA test (*p* < 0.05). Statistical analyses were performed independently for the two treatment periods.

The gradual increase in the levels of hydrogen peroxide (H_2_O_2_) and malondialdehyde (MDA), the two indicators of oxidative stress, in the tomato leaves during salt stress demonstrated the production of secondary oxidative stress in tomato plants under saline conditions. These increases were more pronounced after the 60-day experimental period (Figure 9). Thus, for H_2_O_2_ and in the absence of the biostimulant, increases of ca. 3.8-fold and 7.0-fold were calculated after 30 and 60 days of growth, respectively, when comparing its concentration under the highest (CtrlSSWS) and lowest (CtrlNSWN) salinity conditions tested, reaching a maximum content of 46 µmol g^−1^ DW. The qualitative pattern of variation was very similar for MDA, with 2.6- and 6.2-fold increases calculated at 30 and 60 days, respectively, and reaching a maximum value of ca. 1500 nmol g^−1^ DW (Figure 9).

Treatment with BALOX^®^ reduced the mean values of H_2_O_2_ and MDA contents under all experimental conditions, although this was more clearly observed at higher salinities. To give just a few examples, the maximum H_2_O_2_ concentration measured without biostimulant (CtrlSSWS) was reduced to 32 µmol g^−1^ DW (BaloxSSWS), representing a 30% reduction. Likewise, the maximum MDA concentration value decreased to 1012 nmol g^−1^ DW, a 33% reduction. The differences between the treatments with and without BALOX^®^ were always statistically significant, except for MDA under low salinity conditions (NSWN), in both the 30-day and 60-day treatments (Figure 9).

### 2.7. Antioxidant Enzyme Activities

**Figure 10:**
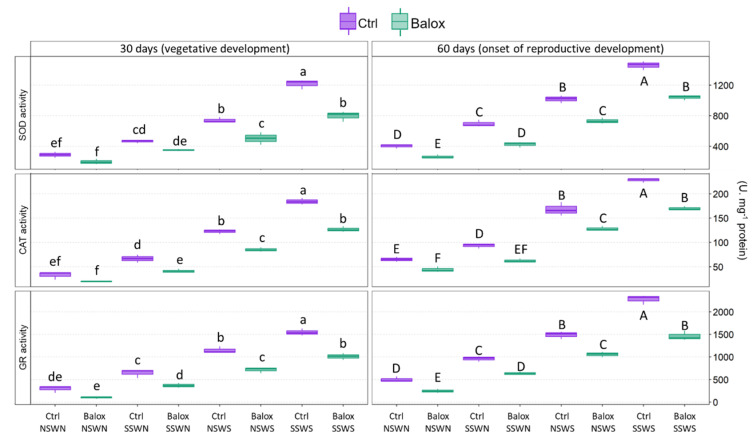
Specific activities of the enzymes superoxide dismutase (SOD), catalase (CAT), and glutathione reductase (GR) in leaf extracts of tomato plants subjected to increasing salinity by different combinations of saline or non-saline soil and irrigation water; the plants were treated, or not, with the biostimulant containing polyphenols at 1.4% (*w*/*w*) and glycine betaine at 3.0% (*w*/*w*) at a dose of 0.4 mL L^−1^ of irrigation water. The control treatments without biostimulant (Ctrl) and the treatments with biostimulant (BALOX^®^) are defined as follows: (i) NSWN: Non-saline soil (EC = 2.28 dS/m) and non-saline water (EC = 0.91 dS/m); (ii) SSWN: Saline soil (EC = 8.55 dS/m) and non-saline water (EC = 0.91 dS/m); (iii) NSWS: Non-saline soil (EC = 2.28 dS/m) and saline water (EC = 10.50 dS/m); (iv) SSWS: Saline soil (EC = 8.55 dS/m) and saline water (EC = 10.50 dS/m). The evaluation periods: 30 days and 60 days of treatment. The values are the means ± SE (*n* = 3). Different letters above the bars (lowercase for 30 days and capital for 60 days of treatments) indicate significant differences between treatments, according to the ANOVA test (*p* < 0.05). Statistical analyses were performed independently for the two treatment periods.

Catalase (CAT), glutathione reductase (GR), and superoxide dismutase (SOD) are some of the most important antioxidant enzymes that plants use to counteract the oxidative stress caused by high salinity conditions; the present study has determined their specific activities in leaf extracts of all harvested plants (Figure 10).

When salt levels increased, the activities of the three enzymes increased significantly and gradually, reaching higher maximum values in samples harvested after 60 days of treatments. For SOD, these values were ca. 1460 units mg^−1^ protein (CtrlSSWS, 60 d) vs. 1220 units mg^−1^ protein (CtrlSSWS, 30 d), and the corresponding activities for CAT and GR were 230 vs. 180 and 2280 vs. 1550 units mg^−1^ protein, respectively (Figure 10).

BALOX^®^ application resulted, in all treatments, in a reduction of the average specific activity values for the three enzymes, which was statistically significant in all circumstances except in the samples harvested after 30 days of treatment under non-saline conditions (NSWN) and in saline soil (SS) for SOD, and in the NSWN for CAT and GR activities. Under the highest salinity conditions (SSWS, 60 days), these reductions amounted to ca. 30%, 25%, and 35% for SOD, CAT, and GR, respectively (Figure 10).

### 2.8. Antioxidant Compounds

In plants, the “second line of defence” against oxidative stress is based on the production of antioxidant metabolites, such as phenolic compounds (especially flavonoids), when activation of antioxidant enzymes alone is insufficient to handle the cellular redox imbalance [43]. Therefore, the leaf levels of total phenolic compounds (TPC) and total flavonoids (TF) were determined in all harvested samples.

**Figure 11:**
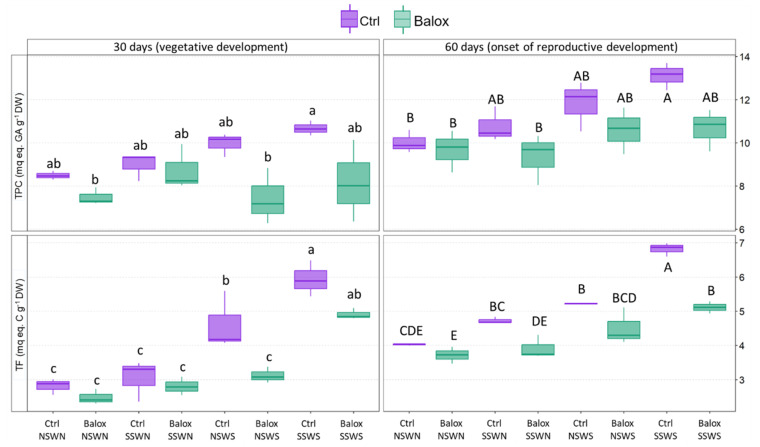
Content of total phenolic compounds (TPC) and total flavonoids (TF) in leaves of tomato plants subjected to increasing salinity by different combinations of saline or non-saline soil and irrigation water; the plants were treated, or not, with the biostimulant containing polyphenols at 1.4% (*w*/*w*) and glycine betaine at 3.0% (*w*/*w*) at a dose of 0.4 mL L^−1^ of irrigation water. The control treatments without biostimulant (Ctrl) and the treatments with biostimulant (BALOX^®^) are defined as follows: (i) NSWN: Non-saline soil (EC = 2.28 dS/m) and non-saline water (EC = 0.91 dS/m); (ii) SSWN: Saline soil (EC = 8.55 dS/m) and non-saline water (EC = 0.91 dS/m); (iii) NSWS: Non-saline soil (EC = 2.28 dS/m) and saline water (EC = 10.50 dS/m); (iv) SSWS: Saline soil (EC = 8.55 dS/m) and saline water (EC = 10.50 dS/m). The evaluation periods: 30 days and 60 days of treatment. The values are the means ± SE (*n* = 3). Different letters above the bars (lowercase for 30 days and capital for 60 days of treatments) indicate significant differences between treatments, according to the ANOVA test (*p* < 0.05). Statistical analyses were performed independently for the two treatment periods.

As observed in Figure 11, the mean values of TPC and TF contents showed a clear increasing trend in response to higher salinities in tomato plants at the vegetative development stage (30-day treatments) and at the beginning of reproductive development (60-day treatments), somewhat more pronounced in the case of TF. However, due to the relatively high variability between biological replicas, reflected in the high SE of the means, these differences were not statistically significant for TPC values and only at high salinity conditions (CtrlNSWS and CtrlSSWS) for TF concentrations. In the latter case, the highest TF content (CtrlSSWS, 60 days) represented a 1.7-fold increase over the low salinity (CtrlNSWN) value. In the Balox-treated plants, a reduction of the mean TPC and TF contents was observed in all treatments, although, as noted above, the alterations were significant only for TF under high salinity conditions in the control plants (Figure 11).

### 2.9. Physiological Trait Relationships and Multivariate Analysis

**Figure 12:**
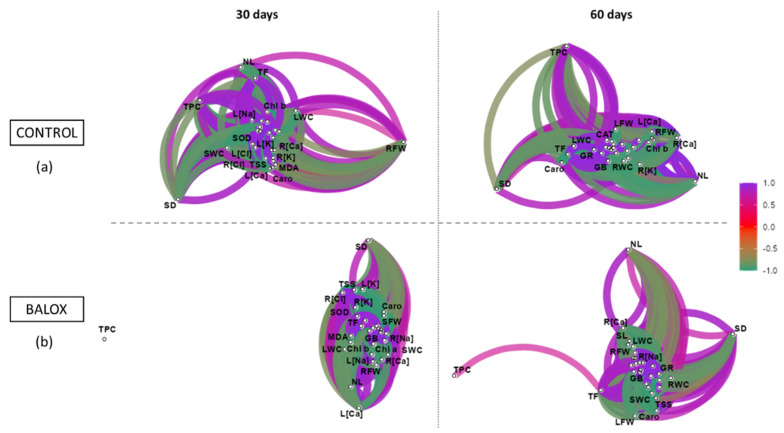
Correlation network plot showing the significant correlations (*p* < 0.05) between the 30 traits measured in each period (30 days: vegetative development; 60 days: onset of reproductive development) in the control (**a**) and plants treated with the biostimulant containing polyphenols at 1.4% (*w*/*w*) and glycine betaine at 3.0% (*w*/*w*) at a dose of 0.4 mL L^−1^ of irrigation water (**b**), according to Pearson’s correlation coefficients. Each measured trait represents a node, and highly correlated traits are grouped. Only significant correlations are represented. Each path represents a correlation between the two variables it links. The purple path represents a positive correlation, and the green path represents a negative correlation. The width and transparency of the line represent the strength of the correlation, so the thicker and more intense the line, the stronger the correlation. Abbreviations: SL: stem length; SD: stem diameter; NL: number of leaves; RFW: root fresh weight; SFW: stem fresh weight; LFW: leaf fresh weight; RWC: root water content; SWC: stem water content; LWC: leaf water content; Chl. a: chlorophyll a; Chl. b, chlorophyll b; Caro, total carotenoids; R [Ca], root calcium; L [Ca], leaf calcium; R [K], root potassium; L [K], leaf potassium; R [Na], root sodium; L [Na], leaf sodium; R [Cl], root chloride; leaf chloride, L [Cl]; Pro, proline; GB, glycine betaine; TSS, total sugars; MDA, malondialdehyde; H_2_O_2_, hydrogen peroxide; SOD, superoxide dismutase; CAT, catalase; GR, glutathione reductase; TPC, total phenolic compounds; and TF, total flavonoids.

A Correlation Network Diagram was performed to identify the degree (positive or negative) and intensity (strong or weak) of the correlations between the 30 variables studied, as shown in Figure 12 and Table S6 of the Supplementary Materials.

On the one hand, a group of strongly and positively correlated variables was observed, including growth parameters such as fresh weight, stem length, and the number of leaves, together with the contents of Chl. a, Chl. b, and Caro, and the concentration of K^+^ and Ca^2+^ ions in roots and leaves. On the other hand, another group of variables that were also strongly and positively correlated with each other was identified, including proline (Pro), MDA, H_2_O_2_, antioxidant enzymes (CAT, SOD and GR), Cl^−^ and Na^+^ concentrations in roots and leaves, and, to a lesser extent, glycine betaine together with the antioxidant metabolites. However, these two groups of observed variables correlated strongly and negatively with each other.

Furthermore, Figure 12a shows that the control plants have activated defence mechanisms such as antioxidant enzymes (SOD, CAT, and GR) and compounds (TPC and TF) at the vegetative stage after 30 days of treatment. The strength of these defence mechanisms increased further at 60 days due to the accumulation of NaCl from saline irrigation water in both non-saline soil (CtrlWS) and saline soil (CtrlSSWS). As has been previously indicated, the first barrier to reducing oxidative stress is activating antioxidant enzymes, which is subsequently followed, if necessary, by the production of antioxidant metabolites such as phenolic compounds. Thus, significant correlations between TPC and other growth-related traits confirm that salinity, under our experimental conditions, generated a considerable level of oxidative stress in control plants..

Unlike the control plants, as shown in Figure 12b, the biostimulant (BALOX^®^) maintains antioxidant enzyme activities below those of the corresponding controls, which means that the plants are less stressed and therefore do not require the activation of a second defence mechanism, especially at 30 days. However, at 60 days, the activation of the synthesis of phenolics is observed, although to a lesser degree than in the control. This attenuating effect is possibly due to the contribution of polyphenols in the formulation of the biostimulant itself and its combination with glycine betaine; therefore, BALOX^®^ could be acting as an antioxidant (polyphenols) and as an osmoprotectant (glycine betaine).

**Figure 13:**
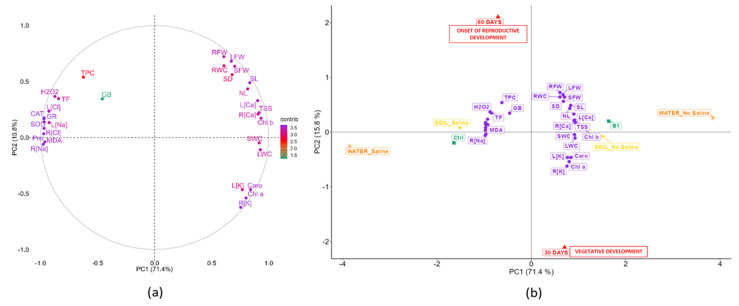
PCA correlation circle (**a**) and PCA biplot (**b**), including the 30 variables analysed in *Solanum lycopersicum* plants subjected to different combinations of soil salinity and irrigation water. Soil salinity (Soil_No Saline: EC = 2.28 dS/m; Soil_Saline: EC = 8.55 dS/m) and water salinity (Water_No Saline: EC = 0.91 dS/m; Water_Saline: EC = 10.50 dS/m), studied at 30 days (vegetative development) and 60 days (onset of reproductive development) in control plants (Ctrl) without biostimulant and in plants treated with biostimulant (B1) containing polyphenols at 1.4% (*w*/*w*) and glycine betaine at 3.0% (*w*/*w*) at a dose of 0.4 mL L^−1^ of irrigation water. The PCAs included, as analysed variables, several growth parameters, photosynthetic pigments, ions, osmolytes, and those related to oxidative stress and antioxidant systems. The first and second principal components explain 71.4% and 15.8% of the total variation, respectively. Symbols: SL, stem length; SD, stem diameter; NL, number of leaves; RFW, root fresh weight; SFW, stem fresh weight; LFW, leaf fresh weight; RWC, root water content; SWC, stem water content; LWC, leaf water content; Chl. a, chlorophyll *a*; Chl. b, chlorophyll *b*; Caro, total carotenoids; R [Ca], root calcium; L [Ca], leaf calcium; R [K], root potassium; L [K], leaf potassium; R [Na], root sodium; L [Na], leaf sodium; R [Cl], root chloride; leaf chloride, L [Cl]; Pro, proline; GB, glycine betaine; TSS, total soluble sugars; MDA, malondialdehyde; H_2_O_2_, hydrogen peroxide; SOD, superoxide dismutase; CAT, catalase; GR, glutathione reductase; TPC, total phenolic compounds; and TF, total flavonoids.

A PCA was performed to examine the relationships between physiological and biochemical parameters measured in control (Ctrl) and BALOX-treated plants (B1), subjected to treatments of increasing salinity by different combinations of saline or non-saline soil and irrigation water (Table S6, Supplementary Materials) during 30 and 60 days, respectively. Figure 13 shows the correlation sphere and the PCA graphic of the 30 parameters.

The 87.2% of the overall variability was covered by the first two components (Figure 13a). The first component (PC1) defined 71.4% of the variability by exhibiting highly significant positive correlations (Figure 13b) with the most important growth parameters: fresh weight and water content of leaves (LFW; LWC), stems (SFW; SWC), and roots (RFW; RWC), the number of leaves (NL), stem length (SL), and stem diameter (SD). Significant positive correlations were also found with chlorophyll levels (Chl. a and Chl. b), total carotenoids (Caro), total sugars (TSS), and K^+^ and Ca^2+^ levels in roots and leaves [R(Ca), R(K); L(Ca), L(K)]. PC1 was negatively correlated mainly with MDA concentration, which showed a strong relationship with proline (Pro) levels and with Na^+^ and Cl^−^ concentrations in roots and leaves. This coincided with the observation that the concentration of MDA and Pro was higher in plants under the salinity (soil and water) conditions to which they were exposed, causing stress and limiting their growth.

The second component (PC2) explained 15.8% of the variability and correlated mainly with factors linked to oxidative stress, such as H_2_O_2_, with the antioxidant systems, especially with the activity of antioxidant enzymes (SOD, CAT, and GR), and with the level of antioxidant compounds (TF and TPC). However, TPC was present to a lesser extent, together with GB. The salt stress treatments (saline water and saline soil) were located in the negative part of the graph, as was the control (Ctrl), together with the parameters related to the inhibition of plant growth. However, the barycenter of the biostimulant is located in the positive part of the axis, which means that it has a protective effect against salinity conditions in the plants, stimulating their growth and allowing them to behave in a similar way to unstressed or less stressed plants, even under high salinity conditions.

## 3. Discussion

In this research, salt treatments on *S. lycopersicum* plants were sufficient to inhibit growth and significantly alter a variety of biochemical markers. These findings were in line with previous studies on the responses of tomato cultivars to different salinity levels [44]. Our results indicate that FW and WC decreased with increasing salinity in the treatments, primarily because of salt accumulation in the soil from irrigation. It is important to draw attention to the FW loss in the samples (CtrlNSWS and CtrlSSWS), which had been watered with saline water, during the 30- and 60-day time frames. However, after 60 days of treatment, an increase in plant growth was observed, which could be justified by the rise in salt resistance with the age of the plants, which are more tolerant as they approach the fruit ripening stage [23]. BALOX^®^ application had an overall positive effect on plant growth and protection against dehydration, as FW and WC increased in the treated plants, especially at 60 days, in all treatments and controls. The stimulation of growth, related to the enhancement of photosynthesis and mineral nutrition, has been reported under the exogenous application of GB and polyphenols, as mentioned in the Introduction. The increase in the total biomass of plants treated with BALOX^®^ in the absence of salt stress is probably related to the enhancement of photosynthetic activity. Plant-based biostimulants frequently promote chlorophyll production and inhibit chlorophyll degradation in stressful environments [45], as reported for tomatoes [46]. In the current study, during both treatment periods, carotenoid and chlorophyll *a* and *b* contents decreased as salinity increased in the absence of the product. However, BALOX^®^ use partly counteracted the salt stress effects, inducing relative increases of the photosynthetic pigments of about two-fold at the highest salinity tested, as well as higher concentrations of these pigments in the control plants. These findings agreed with a prior study [46], which concluded that several bioactive substances, such as glycine betaine, may have contributed to the rise in chlorophyll concentration.

Salt stress adversely impacts the growth and yield of numerous crops through three main mechanisms: generating osmotic stress, the toxicity of Na^+^ and Cl^-^ ions and inducing oxidative stress as a secondary effect [47]. The most prevalent salt, sodium chloride (NaCl), is toxic when present in large quantities [48]. As anticipated, under our experimental conditions, Na^+^ and Cl^−^ levels significantly increased in both roots and leaves of *S. lycopersicum* plants, in parallel with the rise in soil salt content. This increase was even greater in saline irrigation water treatments (NSWS and SSWS) and during the more extended treatment period (60 days). The PCA correlation circle (Figure 13a) showed a negative correlation between fresh weight and water content—two of the most significant growth indices—and the Na^+^ and Cl^−^ concentrations. Additionally, a negative correlation was shown between the levels of the toxic ions and those of photosynthetic pigments, total soluble sugars, and Ca^2+^ and K^+^ in leaves and roots. Our results confirm a previous report indicating the constraining effect of accumulation of the sodium and chloride ions in tomato roots, stems, and leaves, leading to decreases in K^+^, Ca^2+^, and Mg^2+^ levels in all tissues, as well as in chlorophyll and carotenoids [49].

The application of BALOX^®^ favoured plant growth compared to the untreated controls and significantly decreased Na^+^ and Cl^−^ concentrations while increasing those of Ca^2+^ and K^+^ in roots and leaves. The K^+^/Na^+^ ratio significantly increased more than twofold under the highest salinity tested.

Due to the competition between the two cations for the same binding sites, which causes Na^+^ to impede K^+^ uptake through their physiological transport systems, a rise in plant sodium content typically results in a decrease in potassium levels [50]. Na^+^ also causes the plasma membrane to depolarise, which activates outward-rectifying K^+^ channels and results in an extra loss of cellular (vacuolar and cytosolic) K^+^ levels [50,51,52]. This change, in turn, affects chlorophyll content. Consequently, a rise in the Na^+^/K^+^ ratio is typically observed under high salinity conditions, and their balanced ratio is a crucial salt-tolerance mechanism [53]. The main deleterious effect of salinity is the accumulation of sodium and chloride ions in the soil close to the roots, which compromises plant water uptake and reduces the water potential of tissues [54]. By lowering the Na^+^/K^+^ ratio, BALOX^®^ contributed to improving the salt tolerance of the tomato plants.

The plasma membrane H^+^-ATPase is the main mechanism for Na^+^ extrusion in plants [55]. As previously reported [56], qRT-PCR has been used to find out whether GB affects the expression of the genes that encode ion channel proteins in tomato leaves. According to the gene expression data, GB was able to control the H^+^-ATPase during salt stress, improving K^+^ absorption and increasing Na^+^ exclusion, both of which are crucial for maintaining homeostasis. In fact, by limiting Na^+^ buildup in plant tissues and preventing K^+^ loss from the cell, the optimal cytosolic K^+^/Na^+^ ratio may be maintained [57].

Several studies have identified analogous responses on direct and indirect mechanisms enhancing stress tolerance in tomatoes using various biostimulants, including humic acids, fulvic acids, plant-derived hydrolysates, seaweed extracts, and microbial inoculants. For instance, the use of humic and fulvic acids has been associated with the differential regulation of proton pump ATPases in the vacuolar and plasma membranes [58,59], which results in the production of auxin-like compounds and an increase in tomato root development [60,61]. In addition, microbial inoculants have been reported to enhance nutrient uptake, possibly mediated by increases in root biomass, surface area, and root hair number; however, the involved mechanisms are still being elucidated [62]. On the other hand, applications of rhizobacteria promoted *S. lycopersicum* plant growth, increasing fertiliser uptake [63]. In other species, such as maize and wheat, inoculation with Azotobacter strains showed positive effects on salt stress tolerance, facilitating K^+^ uptake and Na^+^ exclusion [64,65]. Growth enhancement by these bacteria has been associated with salt stress attenuation in the presence of high IAA levels [66].

Biostimulants made from seaweed extracts have been reported [67,68] to enhance various plant growth parameters in tomato plants, in addition to increasing chlorophyll levels. In other species [69], microarray analysis was used to assess the effects of an algal extract on gene expression, revealing that the most affected metabolic pathways were those related to photosynthesis, carbon, nitrogen, and sulphur metabolism, and stress responses.

The role of plant-derived hydrolysates has been studied more extensively in the metabolic and morphological modulations of root development in tomato plants [70,71,72]. Phytohormone-like activities have been detected in response to the hydrolysates; in particular, they strongly stimulated the accumulation of cytokinins, auxin, and gibberellin precursors [72]. A protein- and betaine-based biostimulant enhanced the expression of several tomato stress-responsive genes, such as the RAB18 and RD29B orthologues, showing a protective effect on water loss and leading to an increase in fresh weight [73]. It has also been reported that the application of hydrolysates to persimmon trees (*Diospyros kaki* L.) decreased chloride uptake under saline irrigation, increased the concentration of compatible solutes, and improved growth [74]. Several authors [73,75,76] indicated that protein hydrolysates could act directly on nitrogen and carbon metabolic enzymes. In addition, they have auxin and gibberellin-like activities and stimulate antioxidant enzymes and the synthesis of pigments and secondary metabolites. Furthermore, a study by Ozfidan-Konakci et al. [77] showed that phenolic compounds, such as gallic acid, can induce adequate protection against salt stress in rice seedlings.

Similar mechanisms to those mentioned above could be triggered by BALOX^®^ application to reduce the accumulation of Na^+^ and Cl^−^ and, therefore, ion toxicity in the treated plants. Compared to control plants, the relatively lower stress level allows BALOX^®^-treated tomato plants to invest more resources in vegetative development and biomass accumulation.

One of the effects of high salinity and other stressful environmental conditions is the generation of osmotic stress. To maintain osmotic balance, plants respond through the de novo synthesis of organic osmolytes or increasing the transport and accumulation of inorganic ions [54]. Osmolytes, such as proline (Pro), glycine betaine (GB), and several sugars and sugar alcohols, play essential roles in osmotic adjustment. They also have an osmoprotective effect, safeguarding cell structure and function, as well as facilitating water absorption and retention. Many plant species store proline in leaf and root tissues, and it participates in antioxidant systems, neutralises free radicals, and supports cellular redox homeostasis [78,79,80]. There are many reports about Pro accumulation in tomatoes related to the response to salt stress [81,82,83]. Pro content is a good indicator of the degree of stress the plant is experiencing, with numerous studies (e.g., [80]) reporting a robust positive correlation between increased salinity levels and Pro concentrations. This has also been observed in the current investigation, where salt-stressed plants had much higher Pro levels than those of the non-saline treatments. Pro concentrations decreased considerably with the application of BALOX^®^ in comparison to the corresponding controls in both assessment periods, indicating that plants are less stressed. However, this effect cannot be generalised. Other biostimulants, based on microalgae-cyanobacteria [84] or seaweed extracts [85], induced instead a relative *increase* of Pro in plants under salt stress. These data corroborate the ability of some biostimulants to improve the accumulation of osmolytes, mainly due to a high rate of proline synthesis and a reduction in proline dehydrogenase (PDH) activity without degradation of the protein [86].

Under different stress conditions, soluble sugars may also be crucial for osmotic adjustment. Stress-related variations in leaf concentrations of total soluble sugars (TSS) or some particular carbohydrates, such as glucose and myo-inositol, have been observed in numerous crops. (e.g., [87,88]). It has been reported that tomato plants can produce osmotically active organic substances under salt stress conditions, mainly amino acids and sugars, which help to alleviate salinity-mediated osmotic stress [89]. In addition, a study revealed that the higher amount of total sugars in tomato could be responsible for higher salinity tolerance [90]. However, there is not enough information to conclude that sugars play an essential role in plant adaptation to salinity. In addition, the diverse biological functions of sugars as energy sources, components of primary metabolism, or signalling molecules make it difficult to assess their involvement in specific stress response mechanisms; therefore, changes in sugar concentration should be interpreted with caution [91]. In the experiments shown here, the mean TSS values decreased progressively as control plants were subjected to higher salinities and, for each treatment, increased significantly upon BALOX^®^ application, also in the absence of salt. This pattern of TSS changes can be explained by the inhibition of photosynthesis caused by salt and the stimulation observed in the presence of the biostimulant, as indicated by the variation of the photosynthetic pigment contents. In any case, the biostimulant-induced, substantial increase in soluble sugar concentrations should also contribute to osmotic adjustment and, therefore, to salt tolerance. There are reports also showing growth stimulation and increases in photosynthetic pigments and sugar contents in tomatoes by applying other biostimulants based on humic acids [92] or amino acids [93].

Glycine betaine (GB) has also been reported to play several protective roles, including balancing protein and enzyme structures, reducing ROS levels under stress, and maintaining membrane stability under non-physiological circumstances [94]. The outcomes of this work revealed a significant increase in GB levels in parallel with salinity. Previous data [95] showed that the application of GB reduces the effects of abiotic stress in different species. In tomatoes, the GB application significantly improved antioxidant enzyme activities and reduced oxidative stress [56]. In the experiments shown here, contrary to TSS, absolute GB values were too low to be relevant for osmotic adjustment; therefore, any GB effect enhancing salt tolerance should be attributed to its role as an osmoprotectant or free radical scavenger.

During abiotic stress, plants produce more reactive oxygen species (ROS), such as different free radicals and molecules like O_2_, O_3_, or H_2_O_2_ [96]. ROS are regarded as the primary cause of structural damage and are crucial secondary messengers in a variety of physiological processes [97]. Nevertheless, when produced in excess, they become toxic and have a deleterious impact on the cellular metabolism, sometimes even leading to cell death [96]. Growing the tomato plants under conditions of increasing salinity generated oxidative stress as a secondary effect; this was demonstrated by the accumulation of MDA and H_2_O_2_, two reliable biochemical markers, and has been reported in several species exposed to high salinity levels and other stressful environments (e.g., [98,99]). Therefore, a relative reduction of MDA or H_2_O_2_ contents will indicate a lowering of the level of oxidative stress affecting the plants under a particular stressful condition. This was the effect of BALOX^®^ application in the present work, especially in the 60-day treatment. Other authors obtained similar results using a polysaccharide (chitosan) as a biostimulant to counteract salinity stress in tomatoes [100]. The presence of polyphenols as one of the active components in the BALOX^®^ formulation could explain this effect, at least partly. Polyphenols represent a large family of secondary metabolites that are potent antioxidants, complementing the functions of antioxidant vitamins and enzymes. They are involved in maintaining membrane stability (preventing lipid peroxidation) and ROS detoxification; in general, they protect against oxidative stress and contribute to abiotic stress tolerance [101,102].

Several defence mechanisms have been developed by plants to cope with oxidative stress, including activating antioxidant systems. Generally, the first defence reaction involves the activation of enzymes responsible for ROS scavenging: superoxide dismutase (SOD), catalase (CAT), ascorbate peroxidase (APX), and glutathione reductase (GR), amongst others. The activation of these enzymatic systems in plants under salt conditions is well established and contributes to salt tolerance, at least to some extent [103,104,105]. As expected, considering the patterns of variation of MDA and H_2_O_2_ mentioned above, the antioxidant enzyme activities evaluated (SOD, CAT, and GR) increased according to salinity levels and were relatively lower, for each treatment, in plants treated with the biostimulant, which were less stressed than the corresponding controls.

However, in plants subjected to high salinity treatments (CtrlNSWS or CtrlSSWS) and, therefore, to intense oxidative stress, enzyme activation appears insufficient to counteract salt-induced oxidative stress. Under these conditions, a significant increase in endogenous flavonoid levels was detected as a second line of defence. The reduction of the level of oxidative stress in the presence of BALOX^®^ was also reflected in a relative reduction of TF contents. Similar results have been obtained with several extracts also containing polyphenols. For example, *Moringa oleifera* extracts, applied to rocket plants, promoted growth by reducing the level of oxidative stress [106].

Summarising, apart from their effects on ion transport and osmotic adjustment, the bioactive BALOX^®^ components may contribute to lowering the stress caused by elevated salinity by acting as antioxidants (polyphenols) and osmoprotectants (glycine betaine).

## 4. Materials and Methods

### 4.1. Plant Material and Experimental Design

This study was conducted on tomato plants (*Solanum lycopersicum* L. var. DIEZMIL97 F1) in the laboratories and greenhouses of the Polytechnic University of Valencia (Spain), where tomato seeds were sown in trays in the greenhouse with a mixture of commercial peat and vermiculite (1:1). Thirty days after sowing, the seedlings, with an average height of 12 cm, were transplanted into 14-cm-diameter pots filled with 2 kg of soil, either saline or non-saline, as described below. A soil with an electrical conductivity of 2.28 dS/m (NS) was salinised with a 100 mM sodium chloride solution until reaching the saline condition (SS; 8.55 dS/m). Samples were subsequently dried and sieved to 2 mm for physicochemical characterisation of the soil (Table 1).

On 27 April 2021, *Solanum lycopersicum* seedlings were transplanted into individual pots to start the treatments in the greenhouse. With a HOBO U23 Pro v2 data logger (Onset Computer Corporation, EEUU), average temperature (20 °C) and average relative humidity (62%), were monitored over a photoperiod of 16 h per day. The experiment followed a design with multiple variables, consisting of two soil salinities: non-saline (NS) and saline (SS); and two types of irrigation water: non-saline (WN; 0.91 dS/m) and saline (WS; 10.50 dS/m); the latter was obtained by adding 100 mM NaCl to tap water. Soil salinity (NS and SS) combined with irrigation water salinity (WN and WS) generated four levels of progressively increasing salinity: (i) NSWN: non-saline soil and non-saline water; (ii) SSWN: saline soil and non-saline water; (iii) SNWS: non-saline soil and saline water; and (iv) SSWS: saline soil and saline water. All treatments were evaluated after 30 days, in the vegetative developmental stage, and after 60 days, at the onset of reproductive development. The conditions described above (i–iv) were repeated with the application of two different formulations of the biostimulant BALOX^®^ to the plants: **B1**, with 1.4% (*w*/*w*) of total polyphenols expressed as gallic acid equivalents and 3.0% (*w*/*w*) glycine betaine; and **B2**, with the same concentration of total polyphenols and 5.0% (*w*/*w*) glycine betaine. For each treatment, two doses of the biostimulant—0.4 mL L^−1^ (D1) and 0.8 mL L^−1^(D2) —were utilised in addition to the controls (D0), calculated from the doses recommended by the producing company for the use of BALOX^®^ in the field.

The biostimulant is of plant origin, made from hydrolysates of rice and oat husks. BALOX^®^ is applied to the root system through irrigation (alone or in a mixture with fertiliser); it is not intended for use on the leaves.

**Figure 14:**
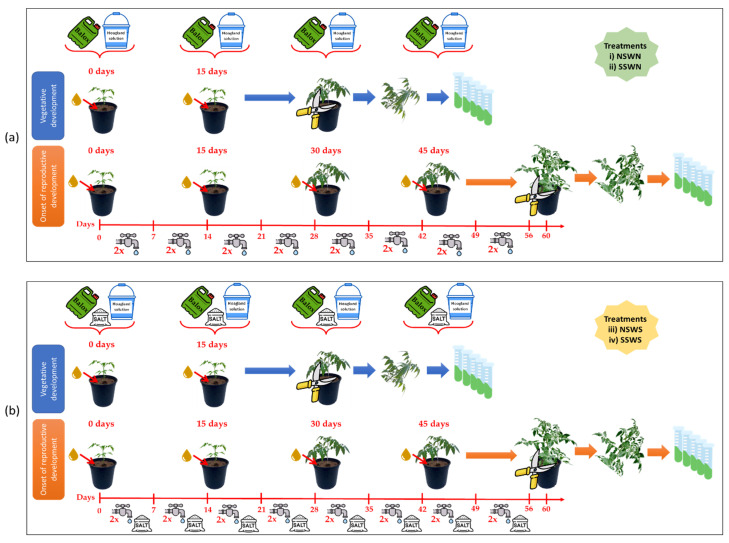
Scheme of the irrigation and biostimulant application on *Solanum lycopersicum* plants subjected to transplantation to different combinations of soil salinity and irrigation water in each period (vegetative development: 30 days; beginning of reproductive development: 60 days). Treatments: (i) NSWN: Non-saline soil (EC = 2.28 dS/m) and non-saline water (EC = 0.91 dS/m); (ii) SSWN: Saline soil (EC = 8.55 dS/m) and non-saline water (EC = 0.91 dS/m); (iii) NSWS: Non-saline soil (EC = 2.28 dS/m) and saline water (EC = 10.50 dS/m); (iv) SSWS: Saline soil (EC = 8.55 dS/m) and saline water (EC = 10.50 dS/m). Two formulations of the biostimulant were applied independently: B1, 1.4% (*w*/*w*) polyphenols and 3.0% (*w*/*w*) glycine betaine, and B2, 1.4% (*w*/*w*) polyphenols and 5.0% (*w*/*w*) glycine betaine. In addition to the controls (D0), two different doses of the biostimulant were also independently applied for each treatment: 0.4 mL L^−1^ (D1) and 0.8 mL L^−1^ (D2). The total number of plants used in the experiments was 120.

Two times each week, 175 mL of non-saline tap water was used to irrigate the plants (i) (NSWN) and (ii) (SSWN) treatments (Figure 14a), and 175 mL of saline tap water for the (iii) (NSWS) and (iv) (SSWS) treatments (Figure 14b). During the first 30 days (vegetative stage), half of the plants treated with biostimulants received two applications of BALOX^®^, directed to the root system through irrigation. Upon transplantation, the first application was carried out, and the second one was made 15 days later. After 30 days, all plants in this batch were harvested (Figure 14). The other half received four applications of BALOX^®^: 0 (transplanting day), 15, 30, and 45 days after the beginning of the experiment. Plants were harvested at 60 days, at the beginning of reproductive development (Figure 14). The schedule of BALOX^®^ applications followed the manufacturing company’s recommendations. In the present work, the biostimulant was prepared in standard Hoagland solution [107] and applied once every 15 days, starting from the day of transplanting. The amount of nutrient solution used per plant was 175 mL with the addition of 100 mM NaCl to the solution for the SNWS and SSWS treatments (Figure 14b); however, for the NSWN and SSWN treatments, NaCl was not added (Figure 14a). At the same time, the control plants also received Hoagland solution in the same way as the treatments but without the biostimulant.

After finishing the experiments, all the variables of the different treatments were evaluated (Figure 1). After observing that both compositions of the biostimulant had very similar effects [B1: polyphenols at 1.4% (*w*/*w*) and 3.0% (*w*/*w*) glycine betaine; and B2: 1.4% (*w*/*w*) polyphenols and 5.0% (*w*/*w*) glycine betaine] as well as the two used doses (D1: 0.4 mL L^−1^ of irrigation water and D2: 0.8 mL L^−1^ of irrigation water), it was decided to show the results of the B1 composition with the lower dose (D1) compared to the controls.

### 4.2. Soil Analysis

The soil used for the experiment was collected from a cultivated area in Valencia (Spain) and was made up of sedimentary material of alluvial origin with calcareous characteristics, common in the Turia river basin (Table 1). The soil is classified as a typical Xerofluvent [108] with a loamy texture (46% sand fraction, 19% clay fraction, and 35% silt fraction), exchange capacity of 4.72 meq/100 g of soil, and a low level of organic matter (0.30%).

### 4.3. Growth Parameters

At the end of the treatments, the number of leaves (NL), stem length (SL), and stem diameter (SD) of all plants were measured. Afterwards, leaf fresh weight (LFW), stem fresh weight (SFW), and root fresh weight (RFW) were determined by gravimetry in a balance. A fraction of fresh leaf material was deep-frozen in liquid nitrogen and stored at −75 °C to be used later in the biochemical assays. The remaining leaf, stem, and root fresh material was weighed and dried at 65 °C until a constant weight was reached. Once dry, it was weighed again to calculate the water content of each plant organ separately. The water content of each sample was calculated according to the equation:

WC (%) = [(FW − DW)/FW] × 100

### 4.4. Determination of Photosynthetic Pigments

Chlorophyll *a* (Chl a), chlorophyll *b* (Chl b), and carotenoids (Caro) contents were determined in an 80% acetone extract prepared from 100 mg of fresh leaf material by following a previously published protocol [109]. Photosynthetic pigments were extracted and shaken overnight at 4 °C in the dark. The samples were centrifuged at 13,300× *g* at 4 °C for 10 min, and the absorbance of the supernatant was measured on a spectrophotometer at 470, 646, and 663 nm. Pigment concentrations were calculated using the following formulas [109]:

Chl a (µg mL ^−1^) = 12.21 (A_663_) − 2.81 (_A646_)

Chl b (µg mL ^−1^) = 20.13 (A_646_) − 5.03 (A_663_)

Caro (µg mL ^—1^) = (1000 (A_470_) − 3.27 [Chl a] − 104 [Chl b])/227

Final values were expressed in mg g^−1^ DW.

### 4.5. Ion Contents Determination

Monovalent ions, potassium (K^+^), sodium (Na^+^), and chloride (Cl^−^), and the divalent cation calcium (Ca^2+^), were determined separately in the roots and leaves in plant water extracts. Samples were extracted by incubating 100 mg of ground dried material in 2 mL of deionised water for one hour at 95 °C in a water bath, cooling on ice, mixing overnight in a shaker, and centrifuging them for 10 min at 13,300× *g* [110]. K^+^, Na^+^, and Ca^2+^ contents were determined using a PFP7 flame photometer (Jenway Inc., Burlington, VT, USA); Cl^−^ was measured in an MKII Chloride Analyser 926 (Sherwood, Inc., Cambridge, UK).

### 4.6. Osmolytes Contents

Osmolyte contents were determined in the leaves of tomato plants. Proline (Pro) was quantified following the acid ninhydrin method [111]. First, 50 mg of fresh material was extracted in 3% aqueous sulphosalicylic acid and mixed with acid ninhydrin. Samples were then incubated in a water bath for one hour at 95 °C, cooled on ice, and extracted with two volumes of toluene. After collecting the organic phase, its absorbance was measured at 520 nm, using toluene as a blank. Samples of known Pro concentrations were assayed in parallel to obtain a standard curve. Pro concentrations were expressed as µmol g^−1^ DW.

Total soluble sugars (TTS) were determined as previously described [112]. Fresh plant material (50 mg) was ground and extracted with 80% (*v*/*v*) methanol. After mixing in a rocker shaker for 24 h, the samples were centrifuged at 13,300× *g* for 10 min; the supernatants were collected, and concentrated sulfuric acid and 5% phenol were added. After 20 min of incubation, the absorbance was measured at 490 nm. TTS concentrations were expressed as glucose equivalents, used as the standard (mg eq. glucose g^−1^ DW).

The concentration of glycine betaine (GB) was measured according to a previously described method [113] with some implemented modifications [114]. Fresh plant material (150 mg) was extracted with 1.5 mL of deionized water and shaken for 24 h at 4 °C, followed by centrifugation at 13,300× *g* for 10 min at 0 °C. The recovered supernatant was mixed (1:1) with a 2 N solution of H_2_SO_4_ and incubated on ice for one hour. Then, 125 µL of the sample was mixed with 50 µL of a KI-I_2_ solution (prepared by dissolving 20 g of KI and 15.7 g of I_2_ in 100 mL of deionised water), causing the precipitation of glycine betaine in the form of golden crystals. All other steps were performed in the dark. Tubes with samples were kept in the fridge at 0–4 °C for 16 h and centrifuged at 13,300× *g* for 45 min at 0 °C. The supernatant was carefully extracted, and the glycine betaine crystals were dissolved in 1.4 mL of cold 1,2-dichloroethane. Finally, after keeping the samples under dark and cold conditions for 2.5 h, their absorbance was measured at 365 nm, using 1,2-dichloroethane as the blank. Reaction mixtures with known GB amounts were run in parallel to obtain a standard curve. GB concentration was expressed as µmol g^−1^ DW.

### 4.7. Oxidative Stress Determination

Two biochemical markers, hydrogen peroxide (H_2_O_2_) and malondialdehyde (MDA), were used to assess the level of oxidative stress in the harvested leaf material.

H_2_O_2_ was measured from 50 mg of fresh leaf material extracted with a 0.1% (*w*/*v*) trichloroacetic acid (TCA) solution, followed by centrifuging the extract. The supernatant was mixed (1:1) with potassium phosphate buffer (10 mM, pH 7.0) and (1:2) KI (1 M). The absorbance was measured at 390 nm [115]. Concentrations were calculated against an H_2_O_2_ standard calibration curve and expressed as µmol g^−1^ DW.

MDA was determined in the same methanol extracts used for TTS determination as previously described in [116], with some modifications [117]. Extracts were mixed with 0.5% thiobarbituric acid (TBA) prepared in 20% trichloroacetic acid (TCA) (or with 20% TCA without TBA for the controls), and then incubated at 95 °C for 20 min, cooled on ice to stop the reaction, and centrifuged at 13,300× *g* for 10 min at 4 °C. The absorbance was measured at 440, 600, and 532 nm. The MDA concentrations were calculated using the equations included in [116]. MDA contents were expressed as nmol g^−1^ DW.

### 4.8. Antioxidant Enzyme Activities

The specific activities of the antioxidant enzymes, specifically superoxide dismutase (SOD), catalase (CAT), and glutathione reductase (GR), were determined in leaf protein extracts of all harvested plants.

Crude protein extracts were prepared from fresh leaf material (100 mg) and stored at −75 °C, as previously described [118]. The protein concentration in extracts was determined following the Bradford method [119], using the Bio-Rad reagent and bovine serum albumin (BSA) as the standard.

SOD activity was determined by monitoring the inhibition of nitroblue tetrazolium (NBT) photoreduction at 560 nm [120]; the reaction included riboflavin as the source of superoxide radicals. One SOD unit was defined as the amount of enzyme needed to cause 50% inhibition of the NBT photoreduction under the assay conditions, as described in the original protocol.

CAT activity was quantified following the consumption of H_2_O_2_ added to the extracts by the decrease in absorbance at 240 nm [121]. One CAT unit is the amount of enzyme necessary to decompose 1 mmol of H_2_O_2_ per min at room temperature.

GR activity was assessed as described in [122], following the oxidation of nicotinamide adenine dinucleotide phosphate (NADPH)—the cofactor in the GR-catalysed reduction of oxidised glutathione—by the decrease in absorbance at 340 nm. One GR unit is defined as the amount of enzyme required to oxidise one mmol of NADPH per min at room temperature.

### 4.9. Antioxidant Compounds Determination

Total phenolic compounds (TPC) and total flavonoids (TF) were quantified in the same methanol extract used for TTS and MDA determination. TPC contents were determined by the reaction with the Folin-Ciocalteu reagent [123]. The absorbance of the samples was measured at 765 nm after 90 min of incubation in the dark at room temperature. TPC contents were expressed as equivalents of gallic acid, used as the standard (mg eq GA g^−1^ DW). Quantification of TF followed a protocol [124] based on the nitration by NaNO_2_ of phenolic rings containing a catechol group and reaction with AlCl_3_ under alkaline conditions. The absorbance was measured at 510 nm, and TF contents were expressed in equivalents of catechin, used as the standard (mg eq C g^−1^ DW).

### 4.10. Statistical Analysis

The four experimental factors —biostimulant formulation (2 levels), water salinity (2 levels), soil salinity (2 levels), and biostimulant dose (2 levels) —were combined, resulting in 16 treatments plus four controls (no biostimulant), for a total of 20 treatments (see Table S5 of the Supplementary Materials). Three randomised replicates were arranged for each harvesting time, resulting in 120 pots.

The data collected at each harvesting time, i.e., at 30 and 60 days after stress imposition (DAS), were analysed through a four-way analysis of variance (ANOVA) to evaluate each experimental factor separately. The Tukey’s honestly significant difference (HSD) test at *p* ≤ 0.05 was applied to identify significant differences between treatments.

To detect and better emphasise the main effects of the different experimental variables and simplify the representation of the results, two preliminary PCAs were performed, with all the data collected at 30 and 60 DAS, respectively. This analysis allowed us to identify those treatments causing the same or very similar effects, namely the two biostimulant formulations and the two biostimulant doses. Only the results obtained, at each harvesting time, with the B1 composition (with lower GB contents) and the lower dose (D1) of the biostimulant were considered for further analysis, compared with the corresponding controls without BALOX^®^.

The selected data were subjected to a one-way analysis of variance (ANOVA) and a post-hoc Tukey’s HSD test. The relationships between the traits were assessed through four correlation network analyses, each referring to the data recorded at 30 or 60 DAS, under control conditions or including the biostimulant treatments. The pairwise Pearson’s correlation coefficients were computed, and their significance was tested at α = 0.05. A final comprehensive PCA was performed to reduce the original variables to a smaller number of principal components while preserving as much statistical information as possible. The PCA included all 30 traits measured at 30 and 60 DAS on the 20 main treatments. The harvesting time, biostimulant type, soil salinity, and water salinity, instead, were used as supplementary categorical variables, i.e., variables that were not involved in the computation of the PCs. The Eigen analysis and the *p*-values of the Pearson correlation coefficients between the 30 active variables and each PC are shown in Tables S6 and S7 of the Supplementary Materials.

The statistical analyses were conducted with RStudio, version 4.1.3, using the packages Car [125] and Emmeans [126] for analysis of variance and post-hoc tests, and FactoMineR [127] and Corrr [128] for principal component and correlation analyses. Charts were generated with the ggplot2 packages [129].

## 5. Conclusions

The application of the biostimulant BALOX^®^ had an overall positive effect on the growth of the treated plants, promoting biomass accumulation by stimulating photosynthesis, as indicated by the increase in photosynthetic pigment levels. The BALOX^®^ application also protected the tomato plants against salt-induced dehydration. The biostimulant’s mechanism of action appears to be partly mediated by the control of ion transport, to some extent inhibiting the uptake of toxic ions such as Na^+^ and Cl^−^ and enhancing the accumulation of K^+^ and Ca^2+^, which help counteract Na^+^ toxic effects. BALOX^®^ also contributed to osmotic adjustment in the presence of salt, increasing the leaf levels of soluble sugars with respect to the controls not treated with the biostimulant. In addition, the significant decrease in the concentrations of proline and oxidative stress biomarkers (MDA and H_2_O_2_) indicates that the salt-treated tomato plants were subjected to a lower degree of oxidative stress in the presence of the biostimulant. Consequently, the substantial increase in the specific activities of several antioxidant enzymes observed in parallel to increasing salinity was also reduced in the BALOX^®^-treated plants. Finally, statistical analyses demonstrated that increasing the concentration of glycine betaine in the biostimulant formulation and doubling its dose had no significant effect on the evaluated plants’ growth parameters and biochemical markers. This finding is important from a practical point of view, as it allows recommending the selection of the biostimulant formulation with the low concentration of GB and its application at the lowest dose.

## Figures and Tables

**Table 1:** Physicochemical characterisation of the soil before starting the treatments. The mean values ± SE (*n* = 3) are shown for each soil parameter.

Soil Characteristics	Not Saline (NS)	Saline (SS)
ECe (dS m^−1^)	2.28 ± 0.03	8.55 ± 0.15
EC_1:5_ (dS m^−1^)	0.37 ± 0.01	1.36 ± 0.02
pH (H_2_O)	7.86 ± 0.02	7.74 ± 0.05
Na^+^ (meq/L)	1.21 ± 0.02	4.01 ± 0.02
K^+^ (meq/L)	5.72 ± 0.03	6.34 ± 0.02
Ca^2+^ (meq/L)	0.76 ± 0.02	1.59 ± 0.05
Mg^2+^ (meq/L)	10.74 ± 0.02	26.46 ± 0.02

ECe: electrical conductivity of the saturated soil extract; EC_1:5_ electrical conductivity, soil:water (1:5) extract.

## Data Availability

Data are contained within the article and supplementary material.

## Supplementary Materials

The following supporting information can be downloaded at: https://www.mdpi.com/article/10.3390/plants12051190/s1. Table S1: Numerical values of FW of leaves, stems, and roots of tomato plants treated with biostimulant under four different salinity conditions: NSWN (non-saline soil and non-saline water); SSWN (saline soil and non-saline water); WNWS (non-saline soil and saline water) and SSWS (saline soil and saline water) evaluated at the stage of vegetative development and the beginning of reproductive development. Table S2: Height (cm), diameter (mm), and number of leaves of tomato plants treated with biostimulant in four different salinity conditions: NSWN (non-saline soil and non-saline water); SSWN (saline soil and non-saline water); NSWS (non-saline soil and saline water); and SSWS (saline soil and saline water) evaluated at the stage of vegetative development and the beginning of reproductive development. Table S3: Water content (%) in leaves (LWC), stems (SWC), and roots (RWC) of tomato plants treated with biostimulant under four different salinity conditions: NSWN (non-saline soil and non-saline water); SSWN (saline soil and non-saline water); NSWS (non-saline soil and saline water), and SSWS (saline soil and saline water) evaluated at the stage of vegetative development and the beginning of reproductive development. Table S4: Chlorophyll *a* and *b* levels in tomato plants treated with biostimulant under four different salinity conditions: NSWN (non-saline soil and non-saline water); SSWN (saline soil and non-saline water); NSWS (non-saline soil and saline water), and SSWS (saline soil and saline water) evaluated at the stage of vegetative development and the beginning of reproductive development. Table S5: Simplified scheme of treatments for each evaluation period, vegetative development (30 days), and the onset of reproductive development (60 days). Table S6: Correlation coefficients between the first two PCs (PC1 and PC2) and the variables included in PCA. Table S7: Eigen analysis of PCA between the 30 active variables.
