# Supplementary material for: Use of a Biostimulant to Mitigate the Effects of Excess Salinity in Soil and Irrigation Water in Tomato Plants
Source: Plants (Basel). 2023 Mar 6;12(5):1190. doi: 10.3390/plants12051190 (PMC10005399; doi:10.3390/plants12051190)
Supplement: Supplementary file 1 [file plants-12-01190-s001.zip › plants-2194159-supplementary.pdf]

Table S1: Numerical values of FW of leaves, stems and roots of tomato plants treated with biostimulant under four different salinity conditions.

PERIOD	TREATMENT	LFW (g)		SFW (g)		RFW (g)	
		CONTROL	BALOX	CONTROL	BALOX	CONTROL	BALOX
Day 30	NSWN	19.14 ± 0.90 ab	21.29 ± 0.52 a	9.65 ± 0.36 a	10.19 ± 0.33 a	2.00 ± 0.23 abc	2.74 ± 0.17 a
	SSWN	16.33 ± 0.58 b	18.57 ± 0.81 ab	7.11 ± 0.13 b	7.72 ± 0.15 b	1.59 ± 0.23 cd	2.58 ± 0.20 ab
	NSWS	11.34 ± 0.37 cd	13.02 ± 0.57 c	4.02 ± 0.42 cd	4.94 ± 0.46 c	1.35 ± 0.06 cd	1.91 ± 0.17 bc
	SSWS	8.46 ± 0.62 d	9.56 ± 0.54 d	2.36 ± 0.08 e	3.05 ± 0.25 de	1.02 ± 0.07 d	1.5 ± 0.10 cd
Day 60	NSWN	27.98 ± 0.75 AB	29.22 ± 0.77 A	18.40 ± 0.45 A	19.75 ± 0.04 A	8.62 ± 0.45 BC	11.29 ± 0.87 A
	SSWN	24.12 ± 0.82 C	27.43 ± 0.69 ABC	13.20 ± 0.31 B	14.43 ± 0.37 B	7.56 ± 0.43 C	10.21 ± 0.24 AB
	NSWS	15.91 ± 0.89 D	24.88 ± 0.53 BC	9.11 ± 0.27 C	10.28 ± 0.53 C	2.16 ± 0.17 DE	3.60 ± 0.34 D
	SSWS	11.77 ± 0.57 E	18.64 ± 1.03 D	3.68 ± 0.43 D	4.82 ± 0.44 D	1.11 ± 0.14 E	1.67 ± 0.10 DE

NSWN (non-saline soil and non-saline water); SSWN (saline soil and non-saline water); NSWS (non-saline soil and saline water) and SSWS (saline soil and saline water) evaluated at the stage of vegetative development and beginning of reproductive development.

Table S2: Height (cm), diameter (mm) and number of leaves of tomato plants treated with biostimulant in four different salinity conditions.

PERIOD	TREATMENT	HEIGHT (cm)		STEM DIAMETER (mm)		NUMBER OF LEAVES	
		CONTROL	BALOX	CONTROL	BALOX	CONTROL	BALOX
Day 30	NSWN	51.70 ± 0.45 a	54.00 ± 0.76 a	4.27 ± 0.07 bc	4.83 ± 0.07 a	11.67 ± 0.33 bc	14.33 ± 0.67 ab
	SSWN	44.73 ± 0.99 b	46.20 ± 0.15 b	4.10 ± 0.06 bc	4.47 ± 0.09 ab	11.33 ± 0.33 cd	14.67 ± 0.33 a
	NSWS	29.40 ± 1.11 d	33.63 ± 0.64 c	3.93 ± 0.07 cd	4.50 ± 0.06 ab	9.33 ± 0.33 cd	11.67 ± 1.20 bc
	SSWS	20.83 ± 0.88 e	26.57 ± 0.54 d	3.63 ± 0.09 d	3.97 ± 0.18 cd	8.67 ± 0.33 d	9.00 ± 0.00 cd
Day 60	NSWN	70.33 ± 0.88 B	76.50 ± 0.76 A	4.77 ± 0.20 ABC	5.37 ± 0.09 A	15.67 ± 0.33 AB	17.00 ± 0.58 A
	SSWN	58.00 ± 0.58 C	71.67 ± 1.09 B	4.50 ± 0.06 BCD	5.10 ± 0.20 AB	14.67 ± 0.33 AB	18.00 ± 1.15 A
	NSWS	34.50 ± 0.29 DE	38.33 ± 0.88 D	4.30 ± 0.15 CD	4.73 ± 0.07 ABC	8.67 ± 0.88 CD	12.00 ± 2.08 BC
	SSWS	25.33 ± 1.20 F	30.67 ± 1.17 E	3.83 ± 0.12 D	4.53 ± 0.12 BC	7.33 ± 0.33 D	11.33 ± 0.33 BCD

NSWN (non-saline soil and non-saline water); SSWN (saline soil and non-saline water); NSWS (non-saline soil and saline water) and SSWS (saline soil and saline water) evaluated at the stage of vegetative development and beginning of reproductive development.

Table S3: Water content (%) in leaves (LWC), stems (SWC) and roots (RWC) of tomato plants treated with biostimulant under four different salinity conditions.

PERIOD	TREATMENT	LWC (%)		SWC (%)		RWC (%)	
		CONTROL	BALOX	CONTROL	BALOX	CONTROL	BALOX
Day 30	NSWN	87.89 ± 0.23 ab	88.68 ± 0.65 a	85.03 ± 0.65 ab	86.35 ± 0.54 a	80.85 ± 0.27 b	83.70 ± 0.34 a
	SSWN	86.76 ± 0.42 abc	88.21 ± 0.35 ab	83.44 ± 0.40 bc	85.64 ± 0.69 ab	77.78 ± 0.56 c	80.76 ± 0.46 b
	NSWS	84.93 ± 0.43 cd	86.24 ± 0.03 bc	80.57 ± 0.30 de	83.38 ± 0.14 bc	73.17 ± 0.31 e	77.17 ± 0.48 cd
	SSWS	83.53 ± 0.61 d	84.88 ± 0.49 cd	78.27 ± 0.63 e	81.87 ± 0.27 cd	71.12 ± 0.44 f	75.80 ± 0.20 d
Day 60	NSWN	87.11 ± 0.68 AB	88.12 ± 0.24 A	90.52 ± 0.15 A	92.66 ± 0.55 A	84.54 ± 0.28 BC	87.80 ± 0.97 A
	SSWN	85.08 ± 0.94 BC	87.84 ± 0.57 A	80.27 ± 0.44 C	83.63 ± 0.57 B	80.56 ± 0.23 D	86.61 ± 0.24 AB
	NSWS	83.27 ± 0.33 CD	85.84 ± 0.43 ABC	73.08 ± 0.27 E	78.07 ± 0.60 CD	78.17 ± 0.44 E	85.25 ± 0.36 BC
	SSWS	81.05 ± 0.49 D	83.99 ± 0.45 C	70.56 ± 0.67 F	76.35 ± 0.30 D	76.43 ± 0.34 E	83.99 ± 0.28 C

NSWN (non-saline soil and non-saline water); SSWN (saline soil and non-saline water); NSWS (non-saline soil and saline water) and SSWS (saline soil and saline water) evaluated at the stage of vegetative development and beginning of reproductive development.

Table S4: Chlorophyll a and b levels in tomato plants treated with biostimulant under four different salinity conditions.

PERIOD	TREATMENT	Chl a (mg g^{-1} DW)		Chl b (mg g^{-1} DW)	
		CONTROL	BALOX	CONTROL	BALOX
Day 30	NSWN	14.71 ± 0.06 b	18.03 ± 0.09 a	3.01 ± 0.01 b	3.62 ± 0.01 a
	SSWN	12.48 ± 0.56 c	17.24 ± 0.47 a	2.71 ± 0.00 bc	3.40 ± 0.10 a
	NSWS	9.93 ± 0.07 d	11.46 ± 0.12 cd	2.03 ± 0.04 d	2.63 ± 0.05 c
	SSWS	6.08 ± 0.36 e	9.98 ± 0.57 d	1.76 ± 0.11 d	2.43 ± 0.12 c
Day 60	NSWN	8.02 ± 0.01 C	11.66 ± 0.15 A	3.04 ± 0.01 C	4.48 ± 0.00 A
	SSWN	7.15 ± 0.12 D	9.77 ± 0.17 B	2.84 ± 0.01 D	4.29 ± 0.00 B
	NSWS	4.45 ± 0.06 E	7.29 ± 0.06 D	1.13 ± 0.00 F	3.04 ± 0.00 C
	SSWS	1.45 ± 0.01 G	2.66 ± 0.01 F	0.74 ± 0.01 G	1.89 ± 0.00 E

NSWN (non-saline soil and non-saline water); SSWN (saline soil and non-saline water); NSWS (non-saline soil and saline water) and SSWS (saline soil and saline water) evaluated at the stage of vegetative development and beginning of reproductive development.

Table **S5:** Simplified scheme of treatments for each evaluation period, vegetative development (30 days) and the onset of reproductive development (60 days). There were 20 treatments with three completely randomised replicates for each harvesting season, resulting in 120 pots.

TREATMENT	BIOST. TYPE	WATER SALINITY	SOIL SALINITY	BIOST. DOSE
1	B0	No Saline	No Saline	D0
2	B1	No Saline	No Saline	D1
3	B1	No Saline	No Saline	D2
4	B2	No Saline	No Saline	D1
5	B2	No Saline	No Saline	D2
6	B0	No Saline	Saline	D0
7	B1	No Saline	Saline	D1
8	B1	No Saline	Saline	D2
9	B2	No Saline	Saline	D1
10	B2	No Saline	Saline	D2
11	B0	Saline	No Saline	D0
12	B1	Saline	No Saline	D1
13	B1	Saline	No Saline	D2
14	B2	Saline	No Saline	D1
15	B2	Saline	No Saline	D2
16	B0	Saline	Saline	D0
17	B1	Saline	Saline	D1
18	B1	Saline	Saline	D2
19	B2	Saline	Saline	D1
20	B2	Saline	Saline	D2

Table S6. Correlation coefficients between the first two PCs (PC1 and PC2) and the variables included in PCA.

QUANTITATIVE VARIABLES	PC1		PC2	
	R	P	R	P
**Caro**	0.840665	7.72E-14	-0.46448	8.82E-04
**CAT**	-0.97122	2.61E-30	0.168129	2.53E-01
**Chl a**	0.801078	8.06E-12	-0.53981	7.51E-05
**Chl b**	0.933014	4.76E-22	0.172514	2.41E-01
**GB**	-0.45897	1.03E-03	0.345237	1.63E-02
**GR**	-0.9688	1.63E-29	0.174967	2.34E-01
**H2O2**	-0.87415	4.95E-16	0.366783	1.03E-02
**L[Ca]**	0.903513	1.52E-18	0.329469	2.22E-02
**L[Cl]**	-0.92674	3.49E-21	0.235047	1.08E-01
**L[K]**	0.768661	1.78E-10	-0.46503	8.68E-04
**L[Na]**	-0.92382	8.30E-21	0.133781	3.65E-01
**LFW**	0.672615	1.64E-07	0.681694	9.60E-08
**LWC**	0.926975	3.25E-21	-0.10908	4.60E-01
**MDA**	-0.96247	1.07E-27	-0.03835	7.96E-01
**NL**	0.816377	1.53E-12	0.433963	2.06E-03
**Pro**	-0.97291	6.64E-31	0.03344	8.21E-01
**R[Ca]**	0.906134	8.32E-19	0.209197	1.54E-01
**R[Cl]**	-0.96876	1.68E-29	0.081782	5.81E-01
**R[K]**	0.755077	5.63E-10	-0.62308	2.25E-06
**R[Na]**	-0.97655	2.49E-32	-0.06034	6.84E-01
**RFW**	0.607026	4.78E-06	0.721556	7.20E-09
**RWC**	0.608127	4.55E-06	0.640369	9.50E-07
**SD**	0.679926	1.07E-07	0.561466	3.30E-05
**SFW**	0.701869	2.73E-08	0.636848	1.14E-06
**SL**	0.834187	1.79E-13	0.4888	4.23E-04
**SOD**	-0.96772	3.52E-29	0.138655	3.47E-01
**SWC**	0.916507	6.31E-20	-0.0473	7.50E-01
**TF**	-0.84234	6.17E-14	0.347384	1.56E-02
**TPC**	-0.62488	2.06E-06	0.538579	7.85E-05
**TSS**	0.916463	6.38E-20	0.221447	1.30E-01
**CATEGORICAL VARIABLES**				
**BIOSTIMULANT**	0.1255	1.35E-02	0.008415	5.35E-01
**PERIOD**	0.023257	3.01E-01	0.936963	2.97E-29
**SOIL**	0.106931	2.33E-02	0.001281	8.09E-01
**WATER**	0.691648	2.47E-13	0.013931	4.24E-01

Variables: stem length (SL); stem diameter (SD); number of leaves (NL); fresh root weight (RFW); stem fresh weight (SFW); fresh leaf weight (LFW); root water content (RWC); stem water content (SWC); leaf water content (LWC); chlorophyll a (Chl.a); chlorophyll b (Chl. b); total carotenoids (Caro); root calcium (R [Ca]); leaf calcium (L [Ca]); root potassium (R [K]); leaf potassium (L [K]); root sodium (R [Na]); leaf sodium (L [Na]); root chloride (R [Cl]); leaf chloride (L [Cl]); proline (Pro); glycine betaine (GB); total sugars (TSS); malondialdehyde (MDA); hydrogen peroxide (H2O2); superoxide dismutase (SOD); catalase (CAT); glutathione reductase (GR); total phenolic compounds (TPC); total flavonoids (TF).

Table S7. Eigen analysis of PCA between the 30 active variables.

PC	EIGENVALUE	PERCENTAGE OF VARIANCE	CUMULATIVE PERCENTAGE OF VARIANCE
**PC1**	2.14E+01	71.4499635	71.44996
**PC2**	4.73E+00	15.7633017	87.21327
**PC3**	1.43E+00	4.76844943	91.98171
**PC4**	7.56E-01	2.51878869	94.5005
**PC5**	3.20E-01	1.06785308	95.56836
**PC6**	2.66E-01	0.88685416	96.45521
**PC7**	2.23E-01	0.74182846	97.19704
**PC8**	1.60E-01	0.53254439	97.72958
**PC9**	1.42E-01	0.47219359	98.20178
**PC10**	1.16E-01	0.38510448	98.58688
**PC11**	9.46E-02	0.3152283	98.90211
**PC12**	8.04E-02	0.26790898	99.17002
**PC13**	5.47E-02	0.18236556	99.35238
**PC14**	4.35E-02	0.1450279	99.49741
**PC15**	2.82E-02	0.09387962	99.59129
**PC16**	2.68E-02	0.08947736	99.68077
**PC17**	2.03E-02	0.06755321	99.74832
**PC18**	1.76E-02	0.05881428	99.80714
**PC19**	1.26E-02	0.0419855	99.84912
**PC20**	1.00E-02	0.0333437	99.88247
**PC21**	9.01E-03	0.03004187	99.91251
**PC22**	8.02E-03	0.0267466	99.93925
**PC23**	5.60E-03	0.01867635	99.95793
**PC24**	4.08E-03	0.01360193	99.97153
**PC25**	3.19E-03	0.01063166	99.98216
**PC26**	2.00E-03	0.00668045	99.98884
**PC27**	1.49E-03	0.00497815	99.99382
**PC28**	8.24E-04	0.00274809	99.99657
**PC29**	6.57E-04	0.00219025	99.99876
**PC30**	3.72E-04	0.00123877	100.00000
